# Metal‐Coordinated Supramolecular Self‐Assemblies for Cancer Theranostics

**DOI:** 10.1002/advs.202101101

**Published:** 2021-06-18

**Authors:** Jiating Xu, Jun Wang, Jin Ye, Jiao Jiao, Zhiguo Liu, Chunjian Zhao, Bin Li, Yujie Fu

**Affiliations:** ^1^ Key Laboratory of Forest Plant Ecology Ministry of Education College of Chemistry Chemical Engineering and Resource Utilization Northeast Forestry University Harbin 150040 P. R. China

**Keywords:** bindind ligands, cancer theranostics, coordination, metal ions, supramolecular self‐assembly

## Abstract

Metal‐coordinated supramolecular nanoassemblies have recently attracted extensive attention as materials for cancer theranostics. Owing to their unique physicochemical properties, metal‐coordinated supramolecular self‐assemblies can bridge the boundary between traditional inorganic and organic materials. By tailoring the structural components of the metal ions and binding ligands, numerous multifunctional theranostic nanomedicines can be constructed. Metal‐coordinated supramolecular nanoassemblies can modulate the tumor microenvironment (TME), thus facilitating the development of TME‐responsive nanomedicines. More importantly, TME‐responsive organic–inorganic hybrid nanomaterials can be constructed in vivo by exploiting the metal‐coordinated self‐assembly of a variety of functional ligands, which is a promising strategy for enhancing the tumor accumulation of theranostic molecules. In this review, recent advancements in the design and fabrication of metal‐coordinated supramolecular nanomedicines for cancer theranostics are highlighted. These supramolecular compounds are classified according to the order in which the coordinated metal ions appear in the periodic table. Furthermore, the prospects and challenges of metal‐coordinated supramolecular self‐assemblies for both technical advances and clinical translation are discussed. In particular, the superiority of TME‐responsive nanomedicines for in vivo coordinated self‐assembly is elaborated, with an emphasis on strategies that enhance the accumulation of functional components in tumors for an ideal theranostic outcome.

## Graphical abstract

This review summarizes the recent progress made on metal‐coordinated supramolecular nanomedicines for cancer theranostic by classifying the coordination metal ions in the order of periodic table. Especially, the superiority of the tumor‐responsive nanomedicines for in vivo coordinated self‐assembling cancer theranostic is discussed, with an emphasis on how to realize high nanomedicine accumulation on tumor and therefore ideal theranostic outcome.

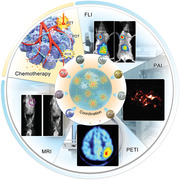

## 1 Introduction

Theranostic nanomedicine, based on a single platform that not only diagnosis but also delivers therapeutics, has emerged as a new discipline owing to the rapid advancement in nanotechnology.^[^ ^1^ ^]^ An appropriately designed nanoplatform that combines a variety of functional components is required for effective theranostics.^[^ ^2^ ^]^ Presently, the most important requirement of the developed nanomedicines for bio‐applications is biosafety; however, the relatively high toxicity and low biodegradability of the majority of nanomedicines have greatly restricted their clinical translation.^[^ ^3^ ^]^ Moreover, solid tumor theranostic nanomedicines face the very intractable problem of low accumulation in tumors.^[^ ^4^ ^]^ Thus, the development of a simple and accurate approach for the design and synthesis of smart theranostic nanomedicines that are both tumor microenvironment (TME) responsive and exhibit enhanced accumulation in target sites is desirable.

To date, numerous cancer theranostic nanomedicines have been constructed via versatile strategies. Nanomedicines comprising covalent bonds are stable during systemic circulation.^[^ ^5^ ^]^ However, drug release at the target sites can be hindered by nanomaterials with excessively high stability. In addition, the complicated and tedious synthesis processes of covalent‐based nanomedicines raise concerns regarding manufacturing, biosafety, drug activity, and hence clinical translation.^[^ ^6^ ^]^ Supramolecular self‐assembly, an approach used to synthesize nanostructures via noncovalent forces, including hydrophobic and electrostatic interactions and hydrogen bonding, is facile and flexible.^[^ ^7^ ^]^ Nevertheless, the complicated in vivo environment may lead to disassembly of these nanostructures because of the unstable nature of the noncovalent bond, thus decreasing drug efficacy and damaging normal tissues. Consequently, the development of theranostic nanomedicines via a simple, robust, and versatile method is required.

Metal‐coordinated self‐assembly, which makes use of the coordination force between metal ions and organic ligands to guide the combination of different organic molecules,^[^ ^8^ ^]^ has recently grown into an elegant strategy to fabricate elaborate theranostic nanosystems by integrating the superiority of both metal coordination interactions and organic self‐assembly. The coordinate covalent bond exhibits both steady and dynamic behaviors in complicated environments because its strength lies between those of weak noncovalent interactions and strong covalent bonds.^[^ ^9^ ^]^ Metal ions and organic ligands can be easily and spontaneously linked by coordination bonds via Lewis acid/base interactions.^[^ ^6a^ ^]^ The key aspects that should be considered when metal‐coordinated self‐assembly is used to develop nanostructures that possess adjustable functionalities and morphologies include metal coordination chemistry, design rules of metal‐binding ligands, selecting rational combinations of various functional components, and control of self‐assembled structures.^[^ ^10^ ^]^

Metal‐coordinated supramolecular nanomedicines are superior to existing nanodrugs because of the following advantages:^[^ ^6^, ^11^ ^]^ i) Metal‐coordinated nanomedicines are constructed at room temperature or under physiological conditions; thus, the synthesis is flexible and environmentally friendly. ii) Diverse dimensions, morphology, and physicochemical properties can be achieved by adjusting the structure and composition of the nanomedicines. iii) Nanomaterials based on coordinate bonds are excellent candidates for safe and smart nanomedicines because of their stability in systematic circulation and precise responsiveness to lesions. iv) A single platform can integrate various properties of the metal ion, organic ligand, and loaded bioactive component.

Metal coordination is used to adjust the self‐assembly routes and to tailor the morphologies of the resulting supramolecular architectures.^[^ ^2^, ^10^ ^]^ The metal ions, along with the binding ligands, determine the properties of the metal‐coordinated supramolecule because various coordination metal ions differ in size, binding affinity, coordination geometry, coordination number, and charge density.^[^ ^10^, ^12^ ^]^ As an example, the small and dense hydration spheres of Mg^2+^ ions are not conducive to ligand coordination, whereas the flexible coordination geometry and unoccupied higher‐energy orbitals of Zn^2+^ ions lead to octahedral and tetrahedral architectures.^[^ ^2^, ^13^ ^]^ Because different metal ions are involved in coordination, the metal–ligand binding affinity, the number of binding ligands, and the orientation of the coordination bond also undergo changes, which facilitates the formation of nanostructures with ideal dimensions, morphology, and physicochemical properties.^[^ ^14^ ^]^ Numerous studies have demonstrated that metal coordination has a significant influence on guiding the self‐assembly of organic ligands into nanomaterials.^[^ ^15^ ^]^ Specifically, the supramolecular self‐assembly of organic ligands can only be driven by coordinating with certain metal ions.^[^ ^16^ ^]^ Overall, metal coordination has become increasingly appealing for the self‐assembly of organic ligands into nanomaterials with different sizes and shapes based on the different geometries and binding stoichiometries of the metal ion.

Metal ions exhibit wide‐ranging electronic, optical, radioactive, magnetic, and catalytic properties, which makes them ideal for imaging and therapy.^[^ ^6a^ ^]^ For instance, Mn^2+^ and Gd^3+^ ions can be utilized in contrast agents for magnetic resonance imaging (MRI) owing to their significant magnetic nature,^[^ ^17^ ^]^ while Fe^2+^ and Cu^+^ can reduce H_2_O_2_ into highly toxic reactive oxygen species (ROS) to realize chemodynamic therapy (CDT).^[^ ^18^ ^]^ Many biomolecules, drug molecules, and pigments that contain metal‐coordinated sites can function as cancer theranostic nanomedicines. However, metal‐coordinated supramolecular self‐assembly can be used to obtain nanomaterials with ideal structures, physicochemical characteristics, and theranostic function. Organic ligands modified with functional molecules can impart additional properties, such as, photodynamic, fluorescent, and photoacoustic effects.^[^ ^19^ ^]^ In addition, some nanomedicines become more stable and biocompatible in the presence of organic ligands.^[^ ^19d^ ^]^ Nanomedicines can be endowed with in vivo targeting and degradable properties through further functionalization.^[^ ^20^ ^]^ Notably, ingenious synthetic methods can produce organic/inorganic nanohybrids that are both TME responsive and degradable to achieve intratumoral self‐assembly permitting the release of the metal ion and organic ligand within the tumor; thus, tumor accumulation of the functional components is enhanced, ultimately improving theranostic results.

**Figure 1:**
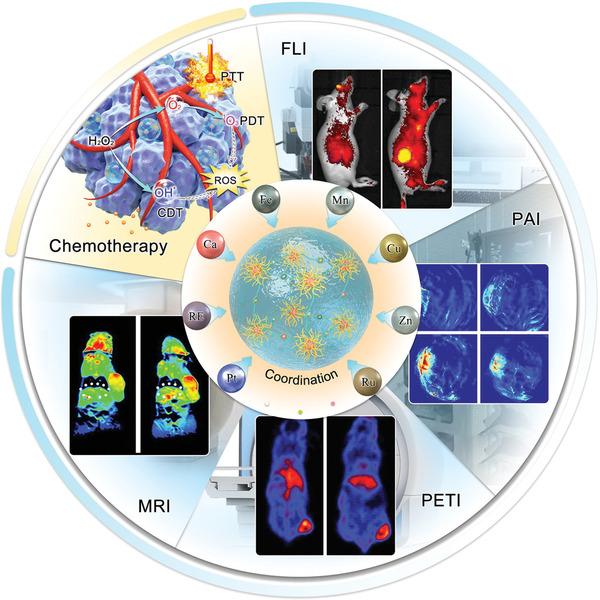
Schematic representation of the metal‐coordinated supramolecular nanomedicines for cancer theranostic (CDT: Chemodynamic therapy, ROS: Reactive oxygen species, FLI: Fluorescence imaging, PAI: Photoacoustic imaging, PETI: Positron emission tomography imaging, MRI: Magnetic resonance imaging). FLI images: reproduced with permission.^[^ ^184e^ ^]^ Copyright 2014, Springer Nature. PAI images: reproduced with permission.^[^ ^30^ ^]^ Copyright 2019, Wiley‐VCH. PETI images: reproduced with permission.^[^ ^39^ ^]^ Copyright 2018, Wiley‐VCH. MRI images: reproduced with permission.^[^ ^37^ ^]^ Copyright 2015, Springer Nature.

To date, a variety of metal‐coordinated supramolecular theranostic nanomedicines have been designed by varying the metal and organic components (**Figure** **1**).^[^ ^21^ ^]^ In this review, we provide a comprehensive outline of recent progress concerning versatile metal‐coordinated self‐assembled nanomaterials for cancer theranostic nanomedicines by classifying these materials according to the coordinated metal ion. The common mechanisms driving the self‐assembly of cancer theranostic nanodrugs comprising different metals, ligands, components, and morphologies are elaborated in detail. We focus on nanomedicines that are TME‐responsive and biodegradable, which are investigated for their tumor‐specific self‐assembly capability to markedly improve their accumulation in tumors and thus the final theranostic outcome. Finally, the challenges and prospects of metal‐coordinated supramolecular nanomedicines are discussed to shed light on cancer theranostic applications.

## 2 Metal‐Coordinated Supramolecular Self‐Assemblies for Cancer Theranostics

As nanotechnology matures, metal‐coordinated nanoassemblies have become increasingly appealing because of their obvious advantages as cancer theranostic nanomedicines (**Table** **1**). First, nanomedicines synthesized using the coordinated self‐assembly strategy can efficiently reduce toxicity in the systemic circulation and improve targeting efficiency, which enhances their accumulation in tumors. Second, metal coordination endows the self‐assembled supramolecular nanomaterial with a variety of exceptional theranostic capabilities, for example, paramagnetic ions are detectable by MRI, and Fenton and Fenton‐like ions cause the CDT effect. Third, the cooperation of various functionalized organic ligands, such as peptides and polyphenols, with different metal ions can form various metal‐coordinated supramolecular nanomedicines with controllable morphology, thus compensating for the drawbacks of many organic anticancer drugs with low drug‐loading content, weak enhanced permeability and retention (EPR) effect, and low biosafety. Presently, research exploration of metal‐coordinated nanomedicines has gradually developed from in vitro self‐assembly to in vivo self‐assembly, as in vivo self‐assembly can achieve more efficient cancer theranostic results. In future, in vivo self‐assembly may become a prominent method for the synthesis of cancer theranostic nanomedicines.

**Table 1:** Typical nanoconstructs prepared by metal‐coordinated supramolecular self‐assembly method for cancer theranostic

Metal ion[s]	Binding ligand[s]	Coordination group[s]	Application[s]	Ref.
Ca^2+^	AS1411 DNA G quadruplexes	Amino and COOH	Drug delivery and cancer theranostic	[22]
Ca^2+^	BP‐KLVFF‐RGD triblock peptide	Amino and COOH	FL imaging and cancer therapy	[23]
V^3+/4+^	Tannic acid	COOH	FL imaging and chemotherapy	[24]
Mn^2+^	Zoledronic acid and DOPA	Phosphate	MR imaging and bisphosphonate delivery	[25]
Mn^2+^	IR825	COOH	MR imaging and PTT	[26]
Mn^2+^	ICG	Sulfonate	FL, MR, PA imaging and PTT	[27]
Mn^2+^	Dithiodiglycolic acid	COOH	MR imaging and drug delivery	[28]
Mn^2+^	Fmoc‐L‐L and Ce6	Amino and COOH	MR imaging and PDT	[29]
Mn^2+^	PheoA and BSA	Amino and COOH	PA imaging and PTT	[30]
Mn^2+^	Biliverdin and Z‐Histidine‐Obzl	Amino and COOH	MR, PA imaging, and PTT	[31]
Mn^2+^	Pheophorbide‐a‐FmocL‐amino acids	Amino and COOH	FL imaging and PDT	[32]
Mn^2+^	Ce6 and ferrocyanide	COOH	FL, MR imaging, and PDT	[33]
Mn^2+^	DVDMS	Amino and COOH	FL, PA, MR imaging, PTT, and PDT	[34]
Mn^2+^	Gallic acid	COOH	FL, MR, PTT, and PDT	[35]
Mn^2+^	Verteporfin	Amino and/or COOH	FL, PA, MR imaging, and PDT	[36]
Fe^3+^	GA and PVP	PhOH and amide	MR imaging and PTT	[37]
Fe^3+^	Bovine serum albumin and GA	PhOH, amino, and COOH	MR imaging and PTT	[38]
Fe^3+^	DOX, platinum prodrug polyphenol, and PEG polyphenol	Amino, COOH, and PhOH	PET imaging, chemotherapy, and CDT	[39]
Fe^3+^	DVDMS and DOX	Amino and COOH	MR imaging, chemotherapy, and PDT	[40]
Fe^2+/3+^	BPDP	COOH	NO therapy and CDT	[41]
Fe^3+^	ICG	Sulfonate	FL, PA imaging, and SDT	[42]
Fe^2+^	DOX and G3139	Amino, COOH, and PhOH	FL, MR iamging, chemotherapy, and gene therapy	[43]
Fe^3+^	EGCG, Pt‐OH, and PEG‐b‐PPOH	PhOH	MR imaging, chemotherapy, and CDT	[44]
Fe^3+^	DOX and EGCG	PhOH	FL, PET imaging, and chemotherapy	[45]
Fe^2+^	Cysteine	Amino and COOH	CDT	[46]
Fe^3+^	Polydopamine	PhOH	FL imaging, Chemotherapy, CDT, and PTT	[47]
Fe^2+^	pTBCB‐PEG	Amino and sulfydryl	PA imaging, CDT, and PTT	[48]
Fe^3+^	DSCP and DOPA	Phosphate and COOH	Chemotherapy and CDT	[49]
Fe^3+^	Sabutoclax and TPEDCC	PhOH and COOH	FL imaging, CDT, and PDT	[50]
Fe^3+^	Hematoporphyrin monomethyl ether	COOH	MR imaging, chemotherapy PDT	[51]
Fe^2+^	PEG‐Ce6 polyphenol and gossypol	PhOH	FL imaging, chemotherapy, PDT, and immunotherapy	[52]
Fe^3+^	Artemisinin	COOH	MR imaging and CDT	[53]
Fe^2+^	4′‐(amino‐methyl phenyl)‐2,2′:6′,2″‐terpyridine modified cyanine	Pyridyl	FL, PA imaging, and CDT	[54]
Fe^3+^	PEG‐polyphenols and DOX	PhOH	MR inaging, CDT, chemotherapy, and immunotherapy	[55]
Fe^2+^	Different polyphenols	PhOH	PTT	[56]
Fe^3+^	Trimesic acid and dopamine	COOH and PhOH	PA, MR imanging, PTT, and CDT	[57]
Fe^3+^	Antisense oligonucleotide and Ribonucleases	Amino and COOH	Co‐delivery of protein and nucleic acid	[58]
Co^2+^	LGAuNPs	Amino	FL imaging	[59]
Cu^2+^	Ferritin protein and DOX	PhOH, amino and COOH	PET imaging and chemotherapy	[60]
Cu^2+^	2‐phenylimidazo [4, 5‐f]‐[1, 10] phenanthroline	Imidazolyl	Cancer therapy	[61]
Cu^2+^	Lcysteine	Amino and COOH	CDT	[62]
Cu^2+^	1,3‐di‐derivative of calix[4]arene	Imidazolyl and PhOH	Cell imaging and cancer therapy	[63]
Cu^2+^	1,3,5‐benzenetricarboxylic acid	COOH	Chemotherapy and CDT	[64]
Cu^2+^	EGCG, ICG, and DOX	Sulfonate and PhOH	FL imaging, chemotherapy, and PTT	[65]
Cu^2+^	CDs‐Ce6	Amino and COOH	FL imaging, PDT, PTT, and CDT	[66]
Cu^2+^	6‐thioguanine	Amino and sulfydryl	MR imaging, chemotherapy, and CDT	[67]
Cu^2+^	[FeII(CN)_6_]	Cyanogen	CDT	[68]
Cu^2+^	DNAzyme and tannic acid	Amino and COOH	CDT and gene therapy	[69]
Zn^2+^	TPZnPc	COOH	FL imaging and PDT	[70]
Zn^2+^	Fmoc‐H and Ce6	Fmoc‐H, imidazole, and COOH	FL imaging and PDT	[71]
Zn^2+^	Fmoc‐H and curcumin	Imidazolyl, COOH, and carbonyl	FL imaging and PDT	[72]
Zn^2+^	Forky peptides	COOH	Chemotherapy	[73]
Zn^2+^	Dipicolylamine and ICG	Pyridyl, imine, and sulfonate	FL, PA imaging, and photo/gene therapy	[74]
Zn^2+^	Nap‐1 and ID‐1 peptide conjugates	Amino and COOH	Drug delivery	[75]
Zn^2+^	H39GFP	Imidazole	FL and MR imaging	[76]
Zn^2+^	TBD‐Br grafted phosphorothiolated DNAzyme	Phosphate	Gene therapy and PDT	[77]
Ru^2+^	2,2′‐biquinoline and 4‐((6‐hydroxyhexyl)oxy)benzonitrile	Pyridyl	PDT and photochemotherapy	[78]
Ru^2+^	Hexa *β*‐Cyclodextrin	Pyridyl	Photochemotherapy	[79]
Ru^2+^	3,5‐bis (4‐(cyanomethyl)phenyl) carbamoyl benzoic acid	COOH	Drug delivery and cancer therapy	[80]
Ru^2+^	1,4‐bis(imidazole‐1‐yl)benzene and 1,3‐bis(imidazol‐1‐yl)benzene	Imidazole	Cancer therapy	[81]
Ru^3+^	2,2′:6′,2′′‐terpyridine (tpy) and 2,2′ biquinoline (biq)	Pyridyl and cyanogen	FL, PA imaging, chemotherapy, and PDT	[82]
Nd^3+^	IR825 and pemetrexed	COOH	FL, PA imaging, chemotherapy, and PTT	[83]
Sm^3+^	(‐)‐epicatechin	PhOH	Cancer therapy	[84]
Gd^3+^	PEI	Amino	FL and MR imaging	[85]
Gd^3+^	DTPA or DOTA	Amino and COOH	MR imaging	[86]
Gd^3+^	Gemcitabine‐50‐monophosphate	Phosphate	MR imaging and chemotherapy	[87]
Gd^3+^	2‐aminoterephthalic acid and cypate	COOH	FL, PA, MR imanging, chemotherapy, and PTT	[88]
Gd^3+^	Rose bengal	COOH and PhOH	FL, MR imaging, PDT, and radiotherapy	[89]
Gd^3+^	RGD‐RFP‐LBT	COOH	FL and MR imaging	[90]
Hf^4+^	c,c,t‐(diamminedichlorodisuccinato)Pt(IV)	COOH	MR imaging and chemoradiotherapy	[91]
Hf^4+^	HI‐4COOH	COOH	PTT	[92]
Hf^4+^	Ce6‐modified polyphenols	PhOH	FL imaging, radiodynamic therapy, and immunotherapy	[93]
Hf^4+^	2,2′‐(((2‐(4′‐(2,2‐dicyano‐1‐phenylvinyl)‐[1,1′‐biphenyl]‐4‐yl)‐2‐phenylethene‐1,1‐diyl) bis(4,1‐phenylene)) bis(oxy)) diacetic acid	COOH	Radiotherapy and radiodynamic therapy	[94]
Pt(II)	Cholesterol	COOH and carbonyl	Chemotherapy	[95]
Pt(II)	mPEG‐b‐PpY	Phosphate	Chemotherapy	[96]
Pt(II)	ALN‐PEG2k‐ALN, ALN‐ASAC8‐PEG2k‐ASAC8‐ALN or ALN‐PEG2k‐ASAC18	Phosphate	Chemotherapy	[97]
Pt(II)	Ad‐terminated poly(aspartic acid)	COOH	FL imaging and chemotherapy	[98]
Pt(IV)	Demethylcantharidin	COOH	CT imaging and chemotherapy	[99]
Pt(II)	Pyridyl	Pyridyl	Drug delivery	[100]
Pt(II)	Pyridine‐functionalized BODIPY	Pyridyl	Chemotherapy and PDT	[101]
Pt(II)	Dipyridyl	Pyridyl	FL imaging and chemotherapy	[102]
Pt(II)	Porphyrin‐containing 120° donor D	Pyridyl	PDT	[103]
Pt(II)	Pyridine derivatives	Pyridyl	Cancer therapy and/or bioimaging	[104]
Pt(IV)	Methylene blue	S and N atom	Chemotherapy and PDT	[105]
Pt(II)	TPC AIEgen	Pyridyl	FL imaging and chemo‐photodynamic therapy	[106]
Pt(II)	Rh‐GFFYERGD	COOH	Chemotherapy	[107]
Pt(II)	120° dipyridyl with a trithioester group	Pyridyl	Drug delivery and chemotherapy	[108]
Fe^3+^ and Cu^2+^	Bovine serum albumin	Amino and COOH	PA imaging and PTT	[109]
Pt(II) and Mn^2+^	5,10,15,20‐tetra(4‐pyridyl)porphyrin and disodium terephthalate	Pyridyl and COOH	FL, MR, PET imaging, and photochemotherapy	[110]
Mn^2+^ and Ru^3+^	Co(C≡N)6 and Ce6	Amino, imidazole, cyanogen, and COOH	MR imaging and PDT	[111]

Thus far, metal‐coordinated supramolecular self‐assembly has been utilized to synthesize diverse nanomedicines for cancer theranostic studies. Because the self‐assembly method is convenient and does not require complicated equipment or rigorous reaction conditions, metal‐coordinated supramolecular nanomedicines have attracted extensive attention in recent years, and considerable research in this field has been reported.

### 2.1 Ca^2+^‐Coordinated Self‐Assemblies

Ca is an important macro‐element in the human body. To date, reports on Ca^2+^‐coordinated supramolecular cancer theranostic nanomedicines are still scarce. The Ca^2+^ ion is present in many biological processes, such as, the construction and self‐assembly of proteins/peptides and their biological effects, in which the Ca^2+^ ion acts as structural elements or cofactors.

#### 2.1.1 In Vitro Coordination Mechanism

**Figure 2:**
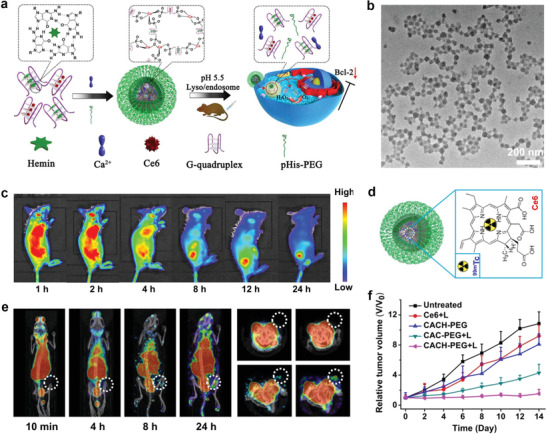
a) Schematic illustration for the synthesis of CACH‐PEG. b) TEM image of CACH‐PEG NCPs. c) In vivo fluorescence images of 4T1‐bearing‐mice taken at different time points after the intravenous injection of CACH‐PEG. d) A scheme illustrating ^99m^Tc‐labeled Ce6 inside CACH‐PEG. e) SPECT images of a representative 4T1‐bearing mouse taken at different time points after the intravenous injection of ^99m^Tc‐CACH‐PEG. f) The tumor growth curves of 4T1‐tumor‐bearing mice after various treatments. Reproduced with permission.^[^ ^22^ ^]^ Copyright 2018, American Chemical Society.

Chen et al. exploited Ca^2+^‐coordinated self‐assembly to prepare DNA‐based nanoscale coordination polymers (NCPs) by mixing Ca^2+^, pHis‐PEG copolymer, and AS1411 G quadruplex in an aqueous solution.^[^ ^22^ ^]^ Both hemin and Ce6 were loaded on the G‐quadruplex structure of AS1411 to obtain Ca‐AS1411/Ce6/hemin@pHis‐PEG (denoted as CACH‐PEG). Importantly, the prepared CACH‐PEG can be used for the intranuclear delivery of Ce6, which efficiently generates ROS inside cell nuclei upon light excitation (**Figure** **2**a). Therein, G‐quadruplex/hemin performs a catalase‐mimicking DNAzyme function; thus, CACH‐PEG can catalyze tumor H_2_O_2_ to O_2_ to alleviate the hypoxic condition during the photodynamic therapy (PDT) process. Moreover, AS1411 can facilitate the down‐regulation of Bcl‐2 expression, thereby improving PDT‐induced cell apoptosis.

The transmission electron microscopy (TEM) image, displayed in Figure 2b, revealed the spherical morphology and narrow size distribution of CACH‐PEG NCPs. Upon intravenous injection of CACH‐PEG NCPs in tumor‐bearing mouse, obvious tumor accumulation was validated by the increased optical signal of Ce6 after 8 h post‐injection, and the intensity of the fluorescence signal in the tumor among all the tested organs peaked at 24 h later, as shown by FL imaging, verifying the efficacious accumulation of CACH‐PEG NCPs in the tumor (Figure 2c). Furthermore, single‐photon emission computed tomography (SPECT) imaging also indicated tumor retention of these NCPs by the EPR effect (Figure 2d,e), which was consistent with the results of FL imaging. Finally, the in vivo PDT effect of these NCPs was studied on 4T1 tumor‐bearing mice. As shown in Figure 2f, compared with all the control groups, the tumors treated with CACH‐PEG NCPs and light irradiation had the lowest tumor‐growth rate.

#### 2.1.2 Bioinspired Coordination Mechanism

Over the last several decades, the in vivo self‐assembly strategy has been promoted to fabricate supramolecular nanostructures for biological applications.^[^ ^112^ ^]^ In particular, physiological stimuli were mostly used to trigger supramolecular interactions that lead to the in situ formation of self‐assemblies for biomedical applications.^[^ ^113^ ^]^ Nevertheless, exploiting metal coordination to adjust the superstructure and morphology of anticancer nanoconstructs in TME still faces significant challenges. In the present section, we summarize the recent works that use metal ions to assemble functional ligands in situ for cancer theranostic applications. Recently, extensive research studies have been focused on developing self‐assembled supramolecular nanostructures that respond to intracellular/intratumoral conditions. Nevertheless, reports on metal‐coordinated intratumoral/intracellular self‐assembled nanomedicines are scarce. In fact, designing TME‐responsive nanomedicines and achieving the intratumoral release of metal ions can induce the rapid supramolecular assembly of released functional ligands. Significantly, these designs are highly promising in markedly improving the intratumoral accumulation of anticancer drugs and ultimately the theranostic outcome.

**Figure 3:**
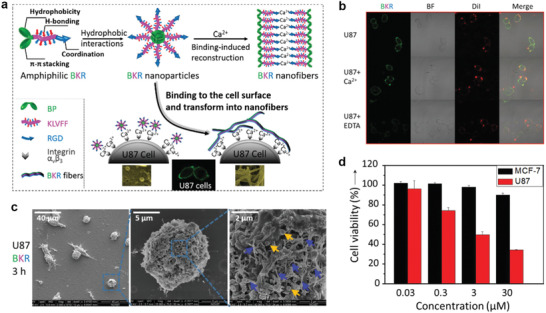
a) Schematic illustration of Ca^2+^‐coordinated reconstruction of BKR nanoassemblies from NPs to nanofibers in solution and on the cell surface. b) SEM images of BKR transformation to nanofibers on U87 cell surfaces. Yellow arrows: Irregular protrusions of cell membranes; red arrows: BKR NPs; blue arrows: BKR nanofibers. c) CLSM images of cells incubated with BKR NPs and the DiI membrane tracker for 10 min. U87 cells (line 1) incubated with BKR NPs for 2 h. Lines 2 and 3 added Ca^2+^ and EDTA‐2Na for 15 min before BKR NPs, respectively. d) Cell viability of U87 and MCF‐7 cells treated with BKR NPs. Reproduced with permission.^[^ ^23^ ^]^ Copyright 2016, the Royal Society of Chemistry.

In 2016, Xu et al. developed an assembly and transformation process to prepare a supramolecular structure with cancer theranostic properties by coordinating Ca^2+^ with BP‐KLVFF‐RGD (BKR) in certain solutions and on specific live cell surfaces (**Figure** **3**a).^[^ ^23^ ^]^ Specifically, the authors synthesized modular peptide‐based building blocks (BKR) which consisted of a divalent cation (Ca^2+^) binding motif (Arg‐Gly‐Asp, RGD), a Lys‐Leu‐Val‐Phe‐Phe (KLVFF) motif, and an aromatic bispyrene (BP) motif. Owing to the strong hydrophobicity and *π*‐*π* stacking interactions, NPs based on BP construct J‐type aggregations in water, which exhibit strong fluorescence. Thus, to promote and observe these aggregations via fluorescence, the authors selected BP as the hydrophobic core. Because RGD naturally binds to integrin *α*v*β*3, Ca^2+^ coordination can regulate the self‐assembly of BKR. KLVFF acted as the peptide scaffold for fiber formation. The KLVFF sequence originating from amyloid *β* segments has been validated to function as a fibrillation motif via intermolecular H‐bonds.

To verify the binding capability of BKR with the Ca^2+^ (*α*v*β*3 integrin) located on the cell surface, U87 cells were employed for the binding experiment of BKR NPs, which indicated that *α*v*β*3 integrin can overexpress. Therefore, BKR NPs were mainly absorbed on the cell membrane (green fluorescence) co‐localized with DiI (red fluorescence) in Figure 3b. The above results suggested that RGD in BKR self‐assembled with Ca^2+^ ions in the v*β*3 integrin to transform into nanofibers in situ from the NPs, which were anchored to the surface of the cell. Additionally, the internalization of BKR NPs in the U87 cells was not enhanced. To further verify that Ca^2+^ can induce the adherence of BKR NPs on cell surfaces, two control groups (Ca^2+^ and EDTA‐2Na pre‐treated U87 cells) were studied. The group pre‐treated with Ca^2+^ exhibited higher fluorescence on the cell surface, which was approximately 1.3 times that of the U87 group (Figure 3b). In contrast, the group pre‐treated with EDTA‐2Na showed lower fluorescence on the cell surfaces, which was ≈0.3 times that of the U87 group, because EDTA‐2Na occupied the Ca^2+^ sites. The above observations were also supported by the scanning electron microscopy (SEM) image of the U87 cells, which were incubated with BKR NPs for 3 h (Figure 3c).

In summary, BKR can self‐assemble into NPs under certain physiological conditions, which can further transform into nanofibers due to its coordination with Ca^2+^. Significantly, the cancer cells can be killed by the peptide nanofibers formed in situ. The BKR “triblock” module can self‐assemble into NPs via hydrophobic interactions and transform into nanofiber in the presence of Ca^2+^ solutions, which can stabilize the H‐bonded KLVFF *β*‐sheets. The BKR NPs can combine with Ca^2+^ ions to illuminate the calcium alginate beads and U87 cells. More importantly, nanofibers can be synthesized by transforming the BKR NPs on the U87 cell membrane to promote cell death. In contrast, the BKR NPs can enter the plasma located in MCF‐7 cells for cell imaging (Figure 3d). Bioinspired morphology transformation of the BKR peptide on cell surfaces plays an important role in cancer theranostics. Furthermore, this work can provide an innovative perspective for transforming superstructures in live cells, thus facilitating the full use of structural changes for cancer theranostics under specific physiological/pathological conditions.

### 2.2 Mn^1+/2+/3+^‐Coordinated Self‐Assemblies

Paramagnetic metal ions generally function as contrast agents to optimize imaging outcomes.^[^ ^25^ ^]^ In particular, metal‐coordinated supramolecular nanoformations that permit the integration of diagnosis and therapy into a single platform are often utilized as cancer theranostic agents, thus fully exploiting the advantages of magnetic metal ions. As an essential trace element of the human body, Mn is a metal element that is closely related to human physiological function. In addition to promoting bone development and maintaining normal physiological function, Mn is also often coordinated with various organic ligands to form supramolecular nanostructures for cancer theranostic.^[^ ^19d^ ^]^

#### 2.2.1 In Vitro Coordination Mechanism

In 2015, Liu et al. fabricated a supramolecular brush polymer as a versatile imaging agent, which was constructed via the self‐assembly of bridged tris(*β*‐cyclodextrin) with Mn^3+^‐porphyrin bonded to poly(ethylene glycol) (PEG) (Mn^3+^‐TPP), and additional functional groups were connected to the supramolecular brush polymer based on the host‐guest interactions of cyclodextrin and adamantine.^[^ ^114^ ^]^ In 2016, Liu et al. developed a new nanoscale metal‐organic particle by coordinating Mn^2+^ ions with IR825 bridging ligands, a near‐infrared (NIR) dye that possesses strong MR imaging quality and excellent photothermal therapy (PTT) efficiency.^[^ ^26^ ^]^ Moreover, a series of Mn‐coordinated nanoscale polymers were developed for cancer theranostic nanomedicines.^[^ ^27^, ^28^, ^91^ ^]^ Various imaging technologies, including X‐ray computed tomography (CT), MRI, ultrasound (US), positron emission tomography (PET), SPECT, and fluorescence (FL) imaging, have matured for cancer diagnosis. However, considering the spatial resolution, sensitivity, and imaging depth, a single imaging technology alone cannot satisfy the high requirements for effective cancer diagnosis. Thus, the development of multimodal imaging techniques and the corresponding contrast agents is necessary.

**Figure 4:**
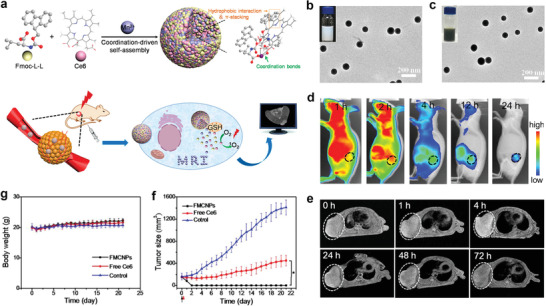
a) Schematic illustration of the fabrication of FMCNPs via coordination‐driven self‐assembly and their responsive disassembly for MRI‐guided PDT. b) TEM images of assembled FMNPs and c) FMCNPs. d) Representative in vivo fluorescence images and e) *T*_1_‐weighted MR images of nude mice bearing MCF7 breast cancer xenografts following the intravenous injection of FMCNPs. f) Change in tumor volume of mice bearing MCF7 breast cancer xenografts treated with FMCNPs and free Ce6 followed by irradiation. g) Variation in the body weight of MCF7 tumor‐bearing mice after various treatments. Reproduced with permission.^[^ ^29^ ^]^ Copyright 2018, American Chemical Society.

In 2018, Yan and coworkers designed a multicomponent coordination supramolecular theranostic platform, which was constructed via the coordination‐driven self‐assembly of Mn^2+^, an amphiphilic amino acid of 9‐fluorenylmethyloxycarbonyl‐L‐leucine (Fmoc‐L‐L), and the photosensitizer chlorin e6 (Ce6) based on the coordination interaction of Mn^2+^ and Fmoc‐L‐L and other noncovalent interactions (*π*‐*π* stacking, hydrophobic interaction) (**Figure** **4**a).^[^ ^29^ ^]^ In the synthetic process, Mn^2+^ was first coordinated with Fmoc‐L‐L to construct Fmoc‐L‐L/Mn^2+^ nanoparticles (FMNPs) (Figure 4b). Subsequently, Fmoc‐L‐L/Mn^2+^/Ce6 nanoparticles (FMCNPs) were obtained by connecting Ce6 molecules to Mn^2+^ by synergistic coordination and other noncovalent interactions (Figure 4c). The fabricated nanomedicines exhibited enhanced accumulation in tumors and improved responsiveness to TME glutathione (GSH), thus achieving the targeted release of imaging and therapeutic agents for excellent theranostic efficacy. The consumption of GSH in the TME was realized through the bonding of Mn^2+^ with GSH. Furthermore, the restriction of ROS generation was alleviated and the retention time of Mn^2+^ was prolonged at the tumor site because of the decrease in GSH content. Therefore, the nanoplatform not only integrated MRI and PDT functions into a single platform but was able to monitor the in vivo delivery of nanomedicines in real‐time to noninvasively evaluate the outcome of therapy.

In vivo experiments in MCF7 tumor‐bearing mice were conducted to determine the distribution of FMCNPs in a living body. The FL intensity and MRI signal were noticeably enhanced at the tumor site after FMCNPs were injected into MCF7 tumor‐bearing mice (Figure 4d,e), which indicated that FMCNPs were accumulated and Ce6 was released at the tumor site, offering real‐time information for imaging‐guided therapy. Furthermore, the PDT effects of the nanomedicines were evaluated. In the three experimental groups, the tumors were successfully suppressed in mice treated with FMCNPs, while the tumor growth in mice injected with free Ce6 was minimally suppressed relative to the control group (Figure 4f). Moreover, the body weight of all the groups remained stable throughout the treatment (Figure 4g). The above results indicated that FMCNPs not only integrated diagnostic and therapeutic functions but also regulated the responsive release of components, affording real‐time information in the imaging‐guided therapy. Overall, FMCNPs are promising as a biocompatible and highly efficient coordinated supramolecular nanomedicine for cancer theranostic applications.

**Figure 5:**
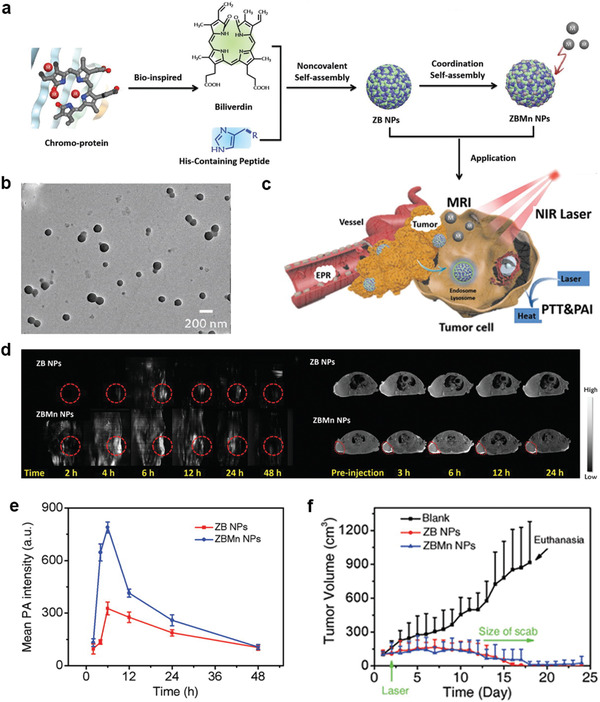
a) Constructing the BV nanoagent by self‐assembly. b) TEM images of the ZBMn NPs. c) The delivery, cellular internalization, and activation of photothermal agents for efficient multimodal imaging and NIR‐excited tumor therapy. d) PAI (left) and *T*_1_‐weighted MRI (right) of tumor‐bearing mice by using the ZB NPs and ZBMn NPs as contrast agents. e) Mean PA intensities acquired from the tumor sites. f) Tumor volume of the mice monitored during the observation period. Reproduced with permission.^[^ ^31^ ^]^ Copyright 2019, Wiley‐VCH.

Recently, photothermal nanomaterials combining PTT and PA imaging (PAI) have attracted widespread attention for cancer theranostic applications. In 2019, Yan et al. constructed photothermal nanomedicines via Mn^2+^‐coordinated supramolecular self‐assembly of proteins and photosensitizers.^[^ ^30^ ^]^ Inspired by the strong coordination of linear tetrapyrrolic pigments with proteins and metals, Yan and coworker constructed biliverdin (BV) nanoparticles (ZBMn) (**Figure** **5**a,b) via the self‐assembly of BV, Z‐Histidine‐Obzl (ZHO), and Mn^2+^.^[^ ^31^ ^]^ The short peptide structure of ZHO comprised an imidazole side chain coordinated with Mn^2+^. BV, an endogenic NIR‐absorbing pigment with well‐studied metabolic pathways, was modulated by the aromatic groups of ZHO. To facilitate coordination interactions and to introduce MRI activity, Mn^2+^ served as the central atom. In vivo data revealed that BV accumulated at the tumor site, and the local temperature increased at the tumor site upon exposure to moderate NIR irradiation, leading to tumor ablation due to the significant photothermal efficiency (Figure 5c). Therefore, the introduction of Mn^2+^ increased the sensitivity of the ZBMn nanosystem for MRI and PAI (Figure 5d,e). As shown in Figure 5f, tumor ablation was observed for the groups treated with ZB and ZBMn NPs. Furthermore, tumor regeneration was not observed during the therapeutic course, while an increase in tumor volume was observed for the blank group. Importantly, Mn^2+^‐coordinated BV NPs exhibited high biosafety; thus, they are promising as a cancer theranostic nanomedicine with multiple functions.

In addition to the above use of Mn^2+^‐coordinated supramolecular nanostructures for imaging‐guided cancer therapy, supramolecular self‐assemblies based on Mn^2+^‐coordination can circumvent the low O_2_ limit in the TME and enhance the efficacy of cancer treatment because of the Mn^2+^‐catalyzed decomposition of tumor endogenous H_2_O_2_ to O_2_.^[^ ^32^, ^115^ ^]^ For example, Yang and coworker constructed a mitochondrial targeting Mn^2+^‐terpyridine (MTP) complex for two‐photon PDT by chelating terpyridine derivates with Mn^2+^; the Mn^2+^ center within MTP catalyzed the conversion of H_2_O_2_ in malignant cancer cells into O_2_ and ROS, which improved the PDT effect during in vivo experiments.^[^ ^116^ ^]^

In a recent report, Zhao and coworkers constructed an antitumor nanomedicine for PDT via multicomponent self‐assembly, in which Prussian blue analogs served as the parent materials.^[^ ^33^ ^]^ The self‐assembly of Mn^2+^ ions, photosensitizers, and organic ligands was achieved based on the coordination bond, and the well‐defined GSH‐depletion nanodrugs was finally protected with biocompatible polyvinylpyrrolidone (PVP). Within the acidic TME, laser‐triggered PDT, GSH depletion, and *T*_1_‐weighted MRI were simultaneously realized. Therefore, all the building blocks were fully utilized and the materials constituting the formation were considered safe by the U.S. Food and Drug Administration, thus ensuring expedited clinical translation. In addition to the abovementioned works, other studies report the utilization of Mn coordination for the synthesis of nanomedicines to achieve theranostic goals.^[^ ^117^ ^]^

#### 2.2.2 Intratumoral Coordination Mechanism

**Figure 6:**
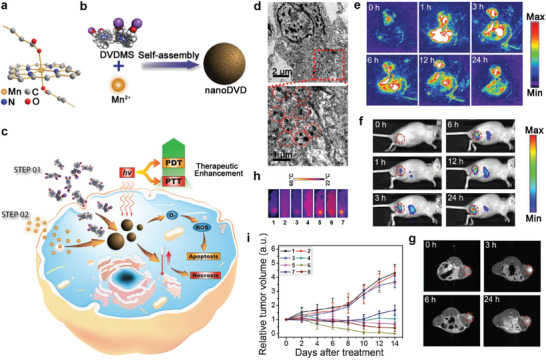
a) Schematic illustration showing a molecular model of the Mn^2+^ linking porphyrin ring and two carboxylate radicals of DVDMS molecules, b) the fabrication process of Mn/DVDMS, and c) PTT/PDT. d) TEM images of MCF‐7 tumor thin sections at 24 h after injection of MnO_2_/DVDMS. e) PA images before and after an intratumoral injection of MnO_2_/DVDMS. f) FL images of the tumor after MnO_2_/DVDMS injection. g) *T*_1_‐weighted MR images before and after injection of MnO_2_/DVDMS. h) Thermal images for different treatments: 1) Saline control + laser; 2) MnO_2_ (i.t.) + laser; 3) DVDMS (i.t.) + laser; 4) DVDMS (i.t.) + Mn^2+^ (i.t.) + laser; 5) MnO_2_/DVDMS (i.t.) + laser; 6) DVDMS (i.v.) + laser; 7) MnO_2_/DVDMS (i.v.) + laser. i) MCF‐7 tumor growth curves of mice treated with different methods: 1) Saline control; 2) MnO_2_/DVDMS (i.t.); 3) saline control + laser; 4) DVDMS (i.t.) + laser; 5) DVDMS (i.t.) + Mn^2+^ (i.t.) + laser; 6) MnO_2_/DVDMS (i.t.) + laser; 7) DVDMS (i.v.) + laser; 8) MnO_2_/DVDMS (i.v.) + laser. Reproduced with permission.^[^ ^34^ ^]^ Copyright 2017, Wiley‐VCH.

In exploring metal‐coordinated in vivo self‐assemblies for cancer theranostic, Chu et al. developed an intriguing intratumoral Mn^2+^‐coordinated self‐assembly of sinoporphyrin sodium (DVDMS) for FL/MR/PA imaging‐guided phototherapy.^[^ ^34^ ^]^ Because of the 3d^5^4s^0^ outermost shell electrons, Mn^2+^ adopted a hexacoordinate structure and thus coordinated with the porphyrin ring and carboxylate radicals on the DVDMS molecule (**Figure** **6**a) to form DVDMS assemblies (Figure 6b). The in vivo phototherapy of Mn^2+^‐coordinated DVDMS nanoassemblies is depicted in Figure 6c. As shown in Figure 6d, when the tumor site was intratumorally injected (i.t.) with MnO_2_/DVDMS, numerous nanoassemblies of DVDMS were clearly observed in the corresponding TEM images of tumor slices. Notably, the integration of FL, MR, and PA imaging in this MnO_2_/DVDMS system was also demonstrated. As shown in Figure 6e, the PA images confirmed that the MnO_2_ content in MnO_2_/DVDMS reduced over time and most of the MnO_2_ disappeared within 24 h after MnO_2_/DVDMS injection. In contrast, the FL signal in the tumor site increased quickly after the injection of MnO_2_/DVDMS (Figure 6f). Additionally, an enhancement in the *T*_1_ MRI signal was observed in the tumor after the injection of MnO_2_/DVDMS (Figure 6g), providing further evidence for the tumor‐responsive release of Mn^2+^ from MnO_2_/DVDMS.

The photo‐induced antitumor effect of the developed MnO_2_/DVDMS was studied with laser irradiation at 630 nm. After MnO_2_/DVDMS was injected, the temperature of the tumor in mice rapidly increased and remained at 45 °C (i.v.) or 50 °C (i.t.) during laser irradiation (Figure 6h). To study the tumor inhibition effect of the developed nanomedicine, changes in tumor volume after different treatments were recorded. As shown in Figure 6i, among the different intratumoral injection treatments, direct drug administration caused an antitumor effect. Notably, MnO_2_/DVDMS (i.t.) achieved greater inhibition of tumor growth than DVDMS (i.t.) combined with Mn^2+^ (i.t.). Additionally, the tumors treated with MnO_2_/DVDMS (i.v.) exhibited a much lower growth rate than those treated with an i.v. DVDMS injection. Therefore, the in vivo Mn^2+^‐coordinated self‐assembly of MnO_2_/DVDMS greatly improved tumor therapeutic efficacy.

In a recent report, an in vivo Mn^2+^‐coordinated supramolecular theranostic nanomedicine,^[^ ^35^ ^]^ comprising MnO_2_, gallic acid (GA), Ce6, hyaluronic acid (HA), and PEG, was developed. Overexpressed tumor GSH and H_2_O_2_ reacted with MnO_2_ loaded on Ce6‐GA@MnO_2_‐HA‐PEG NPs and produced Mn^2+^ and O_2_. The generated O_2_ compromised tumor hypoxia, thereby improving the PDT effect of Ce6‐GA@MnO_2_‐HAPEG NPs in the hypoxic TME. In addition, the released Mn^2+^ ions improved the *T*_1_‐weighted MRI signal. More importantly, the released Mn^2+^ coordinated with GA to form assemblies for PTT. As a result, combinatorial phototherapy significantly inhibited tumor growth. In another study, Tian and coworkers developed an innovative in vivo metal‐coordinated self‐assembly method to extensively embolize the tumor by inducing the coagulation cascade, and the embolization effect was determined using vessel density assessments.^[^ ^36^ ^]^ The authors synthesized a MnO_2_/verteporfin (BPD) nanocomposite, in which MnO_2_ nanosheets served as the carrier to facilitate BPD binding to tumor vessel endothelial cells (TVECs). In the TME, MnO_2_ degraded to Mn^2+^ ions, which coordinated with BPD to form self‐assembled nanoBPD. Compared with free BPD, nanoBPD enhanced TVEC apoptosis and the coagulation cascade. Furthermore, trimodal (MRI/PA/FL) imaging was employed to visualize the tumor vessel density, which was utilized as an indicator to identify patients who would benefit from embolization. These studies provide promising strategies for the eradication of tumors and the prediction of tumor effects, especially in patients with unresectable hepatocellular carcinoma. In summary, intelligent in vivo metal‐coordinated supramolecular nanoconstructs have unparalleled advantages in modulating the TME and improving the accumulation of anticancer components in tumors, thus boosting tumor theranostic outcome.

### 2.3 Fe^2+/3+^‐Coordinated Self‐Assemblies

In the past two decades, the coordination effect between Fe and DNA/RNA has been extensively studied to drive supramolecular self‐assembly of a variety of nanostructures.^[^ ^19^, ^118^ ^]^ Fe is an essential trace metal element of the human body, and Fe^2+/3+^ ions usually participate in diverse metabolic processes, such as, metabolism, immunity, and intracellular redox state regulation. Furthermore, the malignancy of tumors is closely related to Fe^3+^.^[^ ^119^ ^]^ Macrophage differentiation and polarization, by which tumor growth is inhibited or facilitated, are affected by Fe homeostasis. As ROS is produced by the Fe^2+^‐induced Fenton reaction, the intracellular ROS content can increase when the Fe^2+^ content is excessive, resulting in apoptosis of cancer cells, known as ferroptosis.^[^ ^43^, ^120^ ^]^ To date, Fe^2+/3+^ ions have been widely studied as catalysts of the Fenton reaction in CDT, in which Fenton reagents react with endogenous H_2_O_2_ to convert H_2_O_2_ into hydroxyl radicals (·OH) in the TME to kill tumor cells.^[^ ^41^, ^121^ ^]^ The tumor‐suppressing effects of the Fenton reactions have inspired novel approaches for efficacious tumor therapy by introducing exogenous Fe^2+/3+^ ions. Apart from these potential anticancer applications, Fe‐containing compounds can be applied as MRI contrast agents and Fe^2+/3+^ complexes have been validated as excellent candidates for PTT.^[^ ^44^, ^122^ ^]^ Fe has a variety of advantages for biomedical applications owing to its natural abundance and essential role in diverse life processes of the human body.

**Figure 7:**
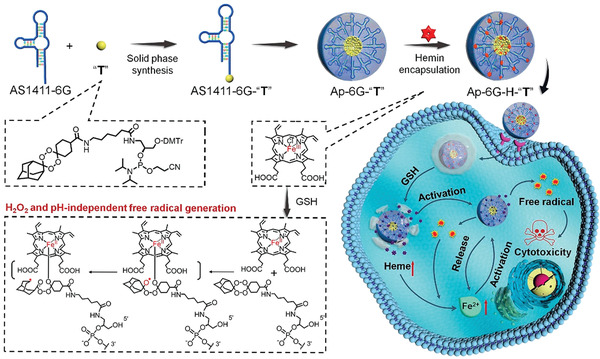
Schematic illustration of bioorthogonal ApPdC micelles for self‐circulation and in situ‐amplified generation of toxic free radical in cancer cells. Reproduced with permission.^[^ ^125^ ^]^ Copyright 2019, American Chemical Society.

Because of the excellent physicochemical and biological characteristics of Fe^2+/3+^ ions, Fe^2+/3+^‐coordinated supramolecular nanohybrids are potential candidates for functional cancer theranostic nanoplatforms.^[^ ^48^, ^51^, ^123^ ^]^ By tailoring the structural components of the organic blocks, various nanoplatforms, such as GA‐Fe@BSA NPs,^[^ ^38^ ^]^ Fe‐DSCP‐PEG‐cRGD,^[^ ^49^ ^]^ and Fe^3+^/ICG@MB,^[^ ^42^ ^]^ with integrated properties have been developed. However, some issues remain unsolved, including limited drug loading, poor circulation stability, low target specificity, limited drug penetration depth across the complicated TME, and low catalytic rate; thus, many researchers have devoted significant efforts to address these problems.^[^ ^124^ ^]^ For example, Xuan et al. combined hemin with adamantane to encapsulate tetraoxane prodrug (T), which was modulated by a nucleotide DNA aptamer AS1411‐6G (Ap‐6G‐H‐2T) (**Figure** **7**).^[^ ^125^ ^]^ To generate toxic C‐centered radicals in situ, cascading bioorthogonal reactions were induced by the intracellular Fe^2+^‐activated prodrug bases in the aptamer prodrug conjugate (ApPdC) micelles. Furthermore, hemin was loaded into the ApPdC micelles via the strongly hydrophobic prodrug bases. Elevating the GSH content in tumor cells reduced the loaded hemin content in heme and therefore generated a Fe^2+^ self‐provided nanosystem that facilitates adequate bioorthogonal reactions without relying on endogenous H_2_O_2_ or strong acidity while decreasing cancerous antioxidation by consuming GSH.

**Figure 8:**
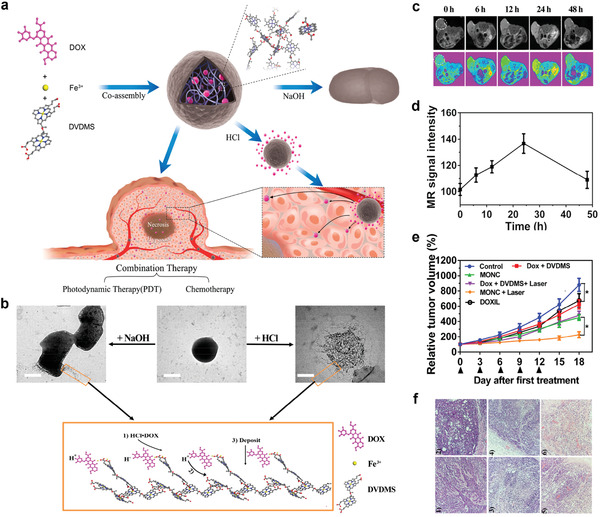
a) Self‐assembly and redelivery of MONCs served as ultrasmall nanodrug generators in response to TME. b) The TEM images of MONC with different proton content are included in the same reaction system and mechanisms of MONC formation, mediated by deposition of deprotonated DOX onto supramolecular scaffold intermediates. c) *T*_1_‐weighted MR images and d) MR signal intensities of tumors (white circles) in mice injected with 6 mg kg^−1^ of MONCs via tail veins. e) Tumor growth profiles and f) representative H&E staining images of tumors in MCF‐7 tumor xenograft mouse model. 1) PBS, 2) DOX + DVDMS, 3) DOX + DVDMS + laser, 4) DOXIL, 5) MONCs, 6) MONCs + laser. Reproduced with permission.^[^ ^40^ ^]^ Copyright 2018, American Chemical Society.

As a typical example, Zhang et al. prepared metal‐organic nanodrug complexes (MONCs) via Fe^3+^‐coordinated supramolecular self‐assembly of the photosensitizer DVDMS and chemotherapeutic drug doxorubicin (DOX).^[^ ^40^ ^]^ The ROS produced by MONCs through energy transfer mediated fluorescence quenching was three times greater than that produced by free DVDMS. Remarkably, the self‐delivering supramolecular MONCs with high drug loading can be utilized as a powerful subminiature drug producer activated by mildly acidic TME, which releases subminiature nanodrugs from larger parental nanoparticles to increase the permeability and therapeutic efficacy (**Figure** **8**a). To adjust the proton content in the MONC formulation, exogenous hydrochloric acid or sodium hydroxide was added into raw MONCs, which had been synthesized without purification at a DVDMS/Fe^3+^/DOX molar ratio of 1:6:10. After separating these nanoparticles, the best MONCs grew into large‐sized cylindrical particles when the proton concentration in the reaction system was decreased. Furthermore, a supramolecular scaffold intermediate was constructed, accompanied by an increase in proton concentration, through the decomposition of partial primary MONCs (Figure 8b). *T*_1_‐weighted MR images and the MR signal intensities of the tumors (white circles) in mice injected with 6 mg kg^−1^ of MONCs via the tail veins were acquired to determine biological safety and *T*_1_‐weighted MRI capabilities (Figure 8c,d). As shown in Figure 8e, tumor inhibition by PEGylated liposomal DOX was suboptimal and comparable to that by DOX. The tumor inhibition effect in mice injected with both DOX and DVDMS and subsequently exposed to laser irradiation was similar to that of the MONC‐treated group; however, the best tumor suppression effect was obtained when the mice were treated with MONCs and laser irradiation. Furthermore, tumors and major organs of the mice were collected 20 days after the first treatment for hematoxylin and eosin (H&E) staining (Figure 8f).

**Figure 9:**
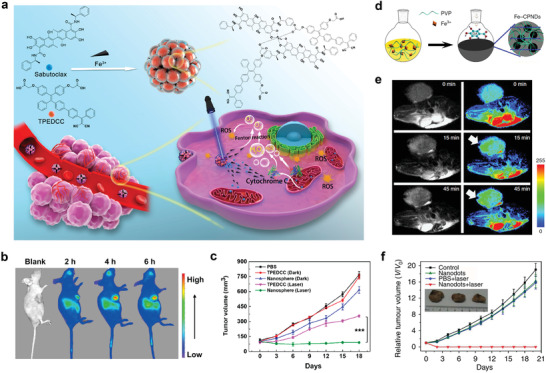
a) The formation of hybrid nanospheres and schematic representation of the hybrid nanospheres taken up by tumor cells, Fe^3+^‐activated Fenton reaction to increase intracellular O_2_ content. Upon 410 nm laser irradiation, TPEDCC produces ROS at low intracellular PDT resistance mitigated by sabutoclax. b) In vivo fluorescence images of MDA‐MB‐231 breast‐tumor‐bearing mice after i.v. injection of the nanospheres. c) Tumor volume change after PBS, TPEDCC and nanosphere treatment with or without laser irradiation in nude mice bearing MDA‐MB‐231. Reproduced with permission.^[^ ^50^ ^]^ Copyright 2020, American Chemical Society. d) The synthesis of Fe‐CPNDs. e) In vivo MR images of nude mice bearing colorectal tumours after intratumoral injection of Fe‐CPNDs at different time intervals (0 min and 0 h indicate pre‐injection). Tumour growth curves of mice in different groups after intravenous treatments. f) The inset shows the digital photographs of tumours collected from different groups of mice at the end of intravenous treatments. Reproduced with permission.^[^ ^37^ ^]^ Copyright 2015, Springer Nature.

In 2020, Shi et al. prepared carrier‐free hybrid nanospheres via Fe^3+^‐coordinated self‐assembly to integrate Fe^3+^, aggregation‐induced emission photosensitizer (TPEDCC), and the Bcl‐2 inhibitor (sabutoclax) into a single nanoplatform (**Figure** **9**a).^[^ ^50^ ^]^ After the nanospheres were intravenously injected into the tumor‐bearing mice, a strong in vivo FL signal was discerned at the tumor site, which indicated that the nanospheres were accumulated in the targeted tumors, and the signal intensity increased to a maximum value after 4 h, which was maintained at 6 h (Figure 9b). Furthermore, in vivo antitumor assays were conducted to study the PDT effect of the nanoplatform, and the experimental results are displayed in Figure 9c. Once the nanospheres were endocytosed by the tumor cells, the Fe^3+^‐triggered Fenton reaction enhances the intratumoral oxygen concentration. Moreover, the sabutoclax and ferroptosis signal pathways relieved the intracellular PDT resistance toward TPEDCC. Therefore, the hybrid nanospheres exhibited high fluorescence with high ROS content generated under laser irradiation, making them ideal candidates for imaging‐guided PDT.

Liu et al. developed a type of pH‐activated nanodots (denoted as Fe‐CPNDs), which were synthesized using coordination reactions among Fe^3+^, GA, and PVP at ambient conditions (Figure 9d).^[^ ^37^ ^]^ Ultrasmall PVP‐protected Fe^3+^‐GA coordination polymer nanodots (Fe‐CPNDs) which were pH‐responsive were constructed via a simple and scalable approach. Due to the ultrasmall hydrodynamic diameter and acidic dissociation nature of the Fe^3+^‐GA_3_ complex, the as‐prepared Fe‐CPNDs easily accumulated at the tumor sites. Additionally, the weakly acidic TME could activate the Fe‐CPNDs, which quickly egressed through the renal system, thus improving the quality of MRI contrast (Figure 9e). As shown in Figure 9f, compared with the other in vivo antitumor groups, the group subjected to the nanodots and laser irradiation presented the highest tumor inhibition effect. These results demonstrated the excellent photothermal conversion effect of Fe‐CPNDs, indicating their promise as PTT agents. Yang et al. developed PVP protected Fe‐quercetin (Qu) coordination nanodrugs (Qu‐FeIIP) via a simple one‐pot synthesis to combine precise diagnosis, excellent low‐temperature PTT efficacy, ROS elimination, and anti‐inflammatory action.^[^ ^120^ ^]^

Recently, materials with strong NIR absorption in the region of 700–3000 nm have attracted extensive attention in the field of NIR photon‐excited phototherapy and optical imaging. This material converts NIR photons, which have high penetration depth in bio‐tissues, into heat for photoacoustic (PA) imaging and thermal ablation of malignant tumors.^[^ ^126^ ^]^ In 2019, Zhang et al. reported a nanoprobe consisting of an upconversion nanoparticle (UCNP) as the core and a coordinatively unsaturated Fe^3+^/GA complex as the shell. In response to the lightly acidic tumor pH, Fe^3+^ in the unsaturated coordination structure was released only in TME. Interestingly, the Fe^3+^ release was quantitatively monitored by comparing the attenuations of multiple upconversion emissions. Moreover, UCNP@GA‐Fe^3+^ can be utilized for cancer therapy by photothermal ablation or a ferroptosis pathway under the guidance of MRI.^[^ ^119a^ ^]^ Similar to NIR photons, ultrasound mechanical waves also have high penetration depths in soft tissues. Thus, ultrasound‐excited treatments can simultaneously achieve relatively high penetration depth and ensure good safety. In this aspect, Zhang et al. constructed a metal‐organic nanosonosensitizer constructed by Fe^3+^‐coordinated self‐assembly of the clinical drug hematoporphyrin monomethyl. Significantly, the Fe^3+^‐coordinated nanoparticles possessed a large surface area and high porosity and thus were used to load DOX. As a result, the developed nanosystem was able to inhibit the growth of deep‐sited tumors through chemo‐sonodynamic therapy.^[^ ^51^ ^]^

**Figure 10:**
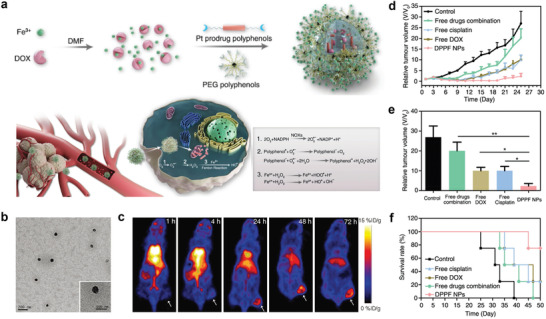
a) Formulation of nanoparticles and the ROS enhanced chemotherapy mechanism. b) TEM images of DPPF nanoparticles. c) Whole‐body PET images of U87MG tumor bearing mouse at 1, 4, 24, 48, and 72 h after intravenous injection of ^89^Zr‐DPPF nanoparticles. d) Tumor volume growth curves and e) the corresponding tumor size with various treatments: DPPF nanoparticles, free DOX, free cisplatin, free drugs combination, and control group (PBS). f) The survival rate curves of U87MG tumor bearing mice with various treatments. Reproduced with permission.^[^ ^39^ ^]^ Copyright 2018, Wiley‐VCH.

In 2018, Dai et al. designed Fe^3+^‐coordinated polyphenol networks to encapsulate DOX and platinum (Pt) prodrugs via a supramolecular self‐assembly process for ROS‐enhanced combination chemotherapy. Therein, both the Pt drugs and DOX can activate nicotinamide adenine dinucleotide phosphate oxidases to generate superoxide radicals (O_2_^•−^). Thereafter, the polyphenols can transform O_2_^•−^ into H_2_O_2_ through their superoxide dismutase‐like property. Further, the highly toxic •OH free radicals were produced by a Fenton reaction (**Figure** **10**a)^[^ ^39^ ^]^ and synergized with the chemotherapy through a cascade of bioreactions. The TEM image displayed in Figure 10b revealed that the size of the DOX@Pt prodrug Fe^3+^ (DPPF) nanoparticles was approximately 70 nm. Importantly, PET imaging of ^89^Zr‐labeled DPPF NPs indicated long blood circulation and high accumulation of the developed nanodrugs in tumors (Figure 10c). As a result, the in vivo antitumor assays indicated that DPPF NPs effectively inhibited tumor growth and minimized the adverse effects of the loaded anticancer components (Figure 10d–f).

Zhao et al. designed chelating complex ferrouscysteine‐phosphotungstate nanoparticles as CDT nanoagent. By introducing phosphotungstate and cysteine, CDT effects were ensured at both neutral and acidic pH, thus circumventing the limited CDT effect in neutral pH.^[^ ^46^ ^]^ Other studies on Fe‐coordinated supramolecular nanoconstructs can be divided into two categories: Fe‐coordinated natural products^[^ ^45^, ^52^, ^53^, ^54^, ^56^, ^58^, ^127^ ^]^ and nanoscale polymer systems.^[^ ^47^, ^55^, ^57^, ^128^ ^]^ All these works demonstrated the advantages and immense potential of Fe^2+/3+^ coordination in constructing multifunctional supramolecular nanodrugs for cancer theranostic applications.

### 2.4 Cu^2+^‐Coordinated Self‐Assemblies

**Figure 11:**
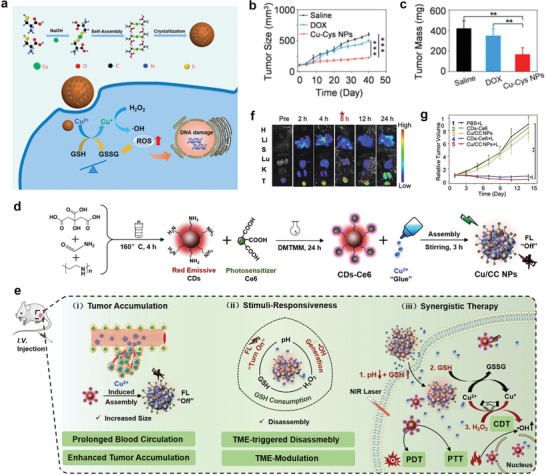
a) Schematic of the Cu‐Cys NPs synthetic process and the Cu‐containing nanoformulation mediated CDT. b) Tumor size change during therapy course. c) Average tumor mass excised from the MCF‐7R tumor‐bearing mice after treatment. Reproduced with permission.^[^ ^62^ ^]^ Copyright 2019, American Chemical Society. d) Illustration of the synthetic process of Cu/CC nanoassemblies, and e) their features for enhancing tumor accumulation, TME stimuli‐responses and synergistic therapy. f) FL images of major organs (H: Heart, Li: Liver, S: Spleen, Lu: Lung, and K: Kidneys) and tumors (T) excised from mice before and after i.v. injection with Cu/CC NPs at various time points. g) Relative tumor volume variation of five treatment groups during the monitoring period. Reproduced with permission.^[^ ^66^ ^]^ Copyright 2020, Wiley‐VCH.

Cu is an essential trace element of the human body. Specifically, Cu can be used to realize chemodynamic cancer treatment, which triggers the generation of highly toxic •OH with the assistance of nanoformulations in the slightly acidic TME.^[^ ^129^ ^]^ For instance, Ma et al. designed a novel self‐assembled copper mercaptide nanoformulation (Cu‐Cys NPs) with a diameter of approximately 80 nm by a facile coordination process between Cu^2+^ ions and the sulfhydryl groups of L‐cysteine in an alkaline solution.^[^ ^62^ ^]^ After the Cu‐Cys NPs enter the tumor cells, local GSH reacts with the Cu‐Cys NPs, inducing GSH consumption and generating Cu^+^ from Cu^2+^ based on the redox reaction. Subsequently, toxic •OH is produced owing to the reaction of local H_2_O_2_ with previously generated Cu^+^ via a Fenton‐like reaction: The reaction rate is fast in the weakly acidic TME, which is responsible for tumor‐cell apoptosis (**Figure** **11**a). Due to the high GSH and H_2_O_2_ contents at the tumor sites, which sequentially induces the redox reactions, the cancer cells were greatly inhibited because of the relatively high cytotoxicity of Cu‐Cys NPs, whereas normal cells were unaffected, which demonstrated the excellent biocompatibility of the Cu‐Cys NPs. Moreover, in vivo results demonstrated that the Cu‐Cys NPs efficiently inhibited the growth of drug‐resistant breast cancer, compared with the effect of an equivalent dose of commercial DOX (Figure 11b,c).

Li et al. constructed a simple Cu^2+^ complex, [CuCl(pip)_2_]Cl, by coordinating Cu^2+^ with 2‐phenylimidazo[4,5‐f]‐[1,10]phenanthroline (pip) in a trigonal pyramidal coordination geometry; the self‐assembly induced the generation of supramolecular metallopolymers via various noncovalent interactions, including *π*‐*π* interactions and hydrogen bonding in acidic solvents.^[^ ^61^ ^]^ After endocytosis into cancer cells, tumor growth in vivo was greatly suppressed by the supramolecular metallopolymers without damage to the major organs. Kong et al. prepared intramolecular Cu‐containing amphiphilic hyperbranched polytriazoles, which subsequently self‐assembled into spherical assemblies because Cu intramolecularly coordinated with the triazole groups in Cu‐catalyzed azide‐alkyne cycloaddition, thus generating copper‐triazole coordination polyprodrugs with a diameter of approximately 50 nm.^[^ ^130^ ^]^ The spherical assemblies were used as fluorescent nanoprobes with aggregation‐induced emission enhancement for cellular bioimaging. Furthermore, an investigation of cell viability showed that Cu was effectively delivered to the tumor sites through the release of the anticancer Cu‐triazole coordination complex by the assemblies.

Although the synthetic polymers used to prepare nanomedicines are biodegradable and biocompatible, they take weeks or months to degrade because the monomers are connected with stable covalent bonds and the final polymers often have relatively high molecular weights. To address this issue, a variety of nanostructures based on diverse natural products have been constructed to promote biomolecular self‐assembly. Peptides, especially short peptides, have considerable advantages in this respect owing to their biocompatibility, easy synthesis, functionality, and adjustable bioactivity.^[^ ^16^, ^131^ ^]^ The self‐assembly of peptide/protein driven by metal ions is an effective strategy for the formation of well‐defined structures for cancer treatment.^[^ ^60^, ^132^ ^]^ The shortcomings of nanostructures connected by noncovalent interactions may be addressed by coordinating metal ions with organic ligands, which facilitates the construction of supramolecular nanostructures with various morphologies.^[^ ^133^ ^]^ Considering the abovementioned points, Reches et al. prepared two short (tri) peptides, L_1_ (Boc‐NH‐Phe‐Gly‐Glu‐OH) and L_2_ (Boc‐NH‐Phe‐Val‐Glu‐OH), with similar backbones but different hydrophobicity of the central amino acid; after coordinating with Cu^2+^, their corresponding conjugates were denoted as L_1_M and L_2_M. Finally, L_1_M self‐assembled into nano‐belt‐like structures, whereas L_2_M self‐assembled into nano‐flake‐like structures by Cu^2+^‐coordinated triggered self‐assembly.^[^ ^134^ ^]^ The experimental results demonstrated that these metallopeptide‐based structures can be used to deliver drugs using a simple and efficient drug displacement strategy.

In a recent study, the self‐assembly of Cu^2+^ and Ce6‐modified CDs (CDs‐Ce6) produced versatile nanoparticles (Cu/CC NPs) that were capable of responding to the TME and consuming GSH (Figure 11d).^[^ ^66^ ^]^ The product possessed the following remarkable features (Figure 11e): 1) FL and photosensitization of Cu/CC NPs occurred in the quenched state under neutral conditions. However, the weak acidity and excessive GSH and H_2_O_2_ contents in the TME selectively activated FL imaging, PDT, and CDT. Thus, side effects in normal tissues were minimized; 2) The reaction between endogenous H_2_O_2_ with Cu^2+^ in the Cu/CC NPs generated highly toxic •OH and Cu^+^, and the generated Cu^+^ consumed GSH significantly. This caused the disassembly of Cu/CC NPs and thus an amplified CDT effect; 3) Tumor penetration, cell uptake, and in vivo clearance benefited from the disassembly of the Cu/CC NPs in the TME; and 4) Under laser irradiation, therapeutic efficacy was significantly enhanced by synergistic CDT, PDT, and PTT because of the intrinsic photothermal and recovered photosensitization capacities of CDs‐Ce6. In vivo experiments validated all the above (Figure 11f,g).

Recently, Liao et al. prepared a type of self‐assembled metallo‐supramolecular nanoflowers with long‐term tumor retention and NIR/acidity‐induced multidrug release for NIR‐II FL imaging‐guided photochemotherapy. In detail, the nanoflowers were constructed by Cu^2+^‐coordinated supramolecular self‐assembly of DOX, indocyanine green (ICG), and (‐)‐epigallocatechin‐3‐gallate through noncovalent interactions (hydrophobic force and *π*‐*π* stacking).^[^ ^65^ ^]^ Under laser irradiation at 808 nm, local photothermal chemotherapy was effectively guided by the stronger NIR‐II fluorescence from the developed nanomedicine, and the nanoflowers effectively killed the tumor cell by synergistic therapeutics. Other good works using Cu coordination to construct theranostic nanoarchitectures have been reported.^[^ ^63^, ^64^, ^67^, ^68^, ^69^, ^135^ ^]^ In summary, because of the superior coordination capability and the inherent chemodynamic nature of Cu ions, Cu‐coordinated nanomedicines are promising in realizing CDT‐based multimodal therapeutic applications.

### 2.5 Zn^2+^‐Coordinated Self‐Assemblies

Zn is an essential trace element of the human body and plays an important role in human health and activities. Specifically, Zn possesses diverse functions, such as, growth and development, retainment of normal appetite, enhancement of immunity, and promotion of wound healing. To date, studies on Zn‐coordinated nanoconstructs can be divided into two main categories: those constructed through coordination between the Zn and N atoms on the imidazole or porphyrin ring^[^ ^136^ ^]^ and through coordination between Zn and the natural products of amino acid/peptide/protein.^[^ ^73^, ^75^, ^76^, ^137^ ^]^

**Figure 12:**
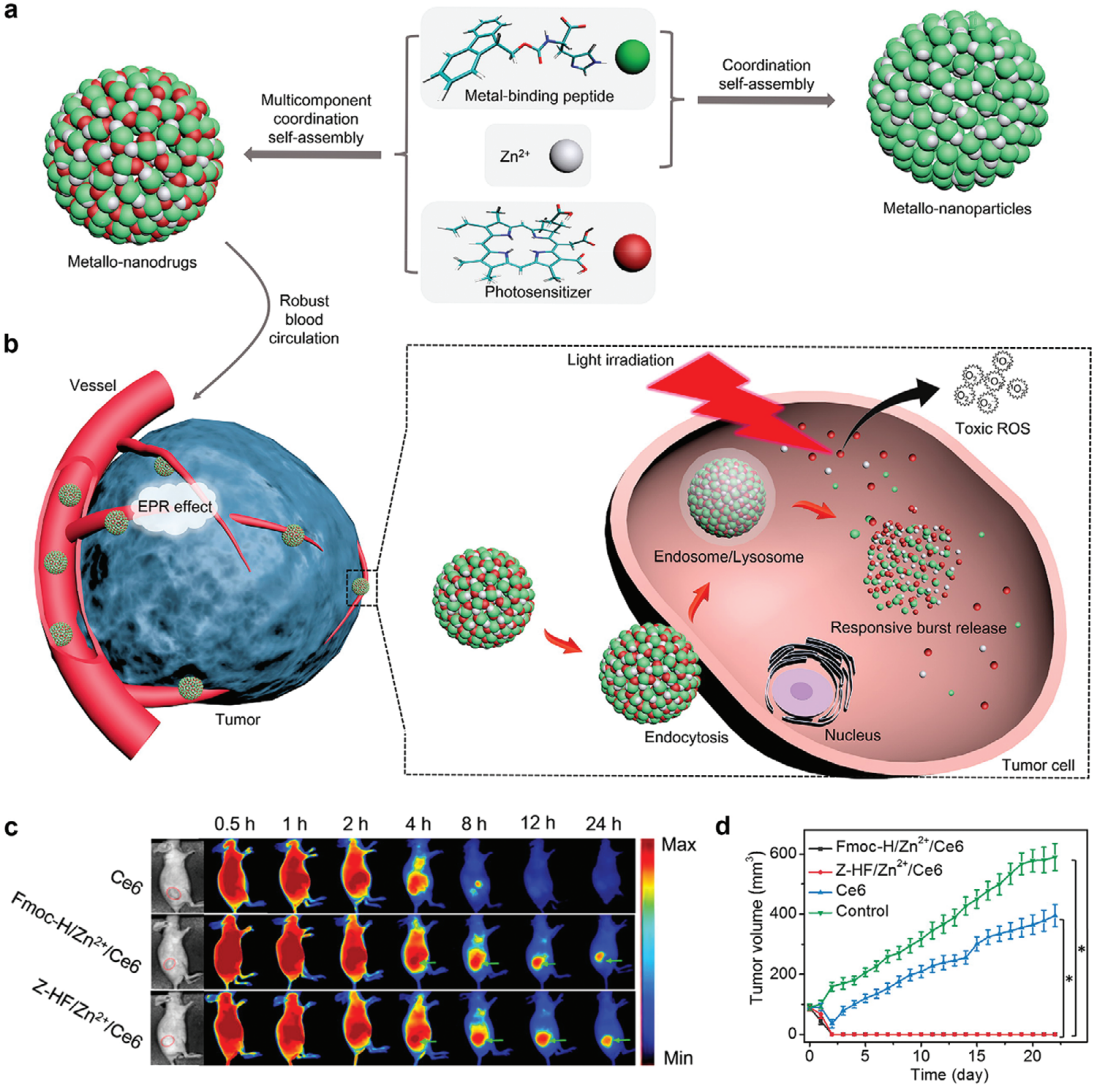
a) Schematic illustration of the preparation of metallo‐nanodrugs through cooperative coordination of small peptides and photosensitizers in the presence of Zn^2+^ ions and b) the supramolecular metallo‐nanodrugs for efficient PDT. c) Fluorescence images showing that Fmoc‐H/Zn^2+^/Ce6 and Z‐HF/Zn^2+^/Ce6 allow better accumulation of Ce6 in tumor sites than unencapsulated Ce6. d) Tumor growth profiles during the observation. Reproduced with permission.^[^ ^71^ ^]^ Copyright 2018, American Chemical Society.

As a typical example, Yan and coworkers developed a versatile and potent multicomponent self‐assembly strategy for PDT nanodrugs. Inspired by the Zn^2+^ ion containing metalloproteins and pigments and the multicomponent self‐organization of polypeptides, FmocH/Zn^2+^/Ce6, a smart metallonanodrug, was synthesized via the cooperation of nonvalent interactions, including hydrophobic and electrostatic interactions, and multiple coordination of Zn^2+^ ions with short peptides and photosensitizers (**Figure** **12**a). The in vivo blood circulation, EPR‐mediated accumulation in tumors, and antitumor therapy of the metallonanodrugs are depicted in Figure 12b. Owing to their well‐defined nanosphere structures, well‐distributed sizes, and high drug encapsulation capabilities, the developed metallonanodrugs achieved prolonged blood circulation and increased accumulation in tumors, which significantly improved the final PDT outcome (Figure 12c,d).^[^ ^71^ ^]^

**Figure 13:**
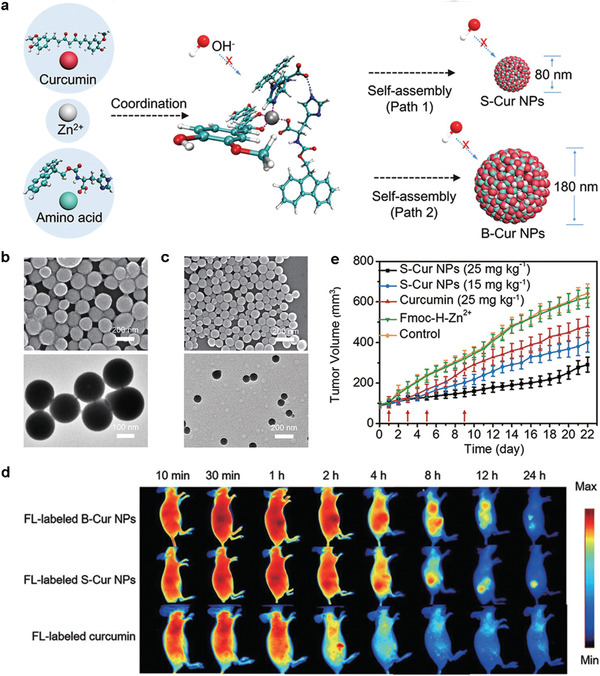
a) Curcumin nanoagents based on Zn^2+^‐coordination driven self‐assembly, and the size of the nanoagents can be rationally controlled to facilitate tumor accumulation. SEM and TEM images of the b) B‐Cur NPs and c) S‐Cur NPs. d) Whole‐body fluorescence images of mice at various time points after the intravenous injection of FL‐labeled B‐Cur NPs, S‐Cur NPs, or curcumin. e) Tumor volumes of the tumor‐bearing mice of different groups after multiple injections at the time points indicated by the red arrows. Reproduced with permission.^[^ ^72^ ^]^ Copyright 2018, Wiley‐VCH.

Yan's group used a Zn^2+^‐coordination‐driven self‐assembly strategy to fabricate a supramolecular curcumin nanoagent. In detail, Zn^2+^ ions, amino acids, and curcumin acted as building blocks to construct well‐defined and uniform curcumin nanodrugs by combining metal coordination and multiple noncovalent interactions.^[^ ^72^ ^]^ Zn^2+^ coordination and molecular stacking protected curcumin from degradation (the attack of hydroxide ion at the diketo/enol moiety). The size distribution of the nanoagent was easily controlled by regulating the self‐assembly kinetics and thermodynamics (**Figure** **13**a). The SEM and TEM images of the big‐sized curcumin nanoparticles (B‐Cur NPs) and small‐sized curcumin nanoparticles (S‐Cur NPs) are displayed in Figure 13b,c, respectively. Two main issues should be resolved before curcumin can be considered for clinical translation: Rapid degradation of this molecule in neutral, physiological media, and low accumulation in tumors. Notably, the stability of curcumin was markedly enhanced with the formation of B‐Cur NPs and S‐Cur NPs. The curcumin in B‐cur NP and S‐cur NP remained respectively at 67% and 77% after a long incubation period of one month. Interestingly, the developed B‐Cur NPs and S‐Cur NPs were stable in normal tissue and activated when the pH was decreased or the GSH content was increased in the TME to rapidly release the loaded curcumin. Furthermore, B‐Cur NPs or S‐Cur NPs selectively accumulated in the cancer cell of mice injected with the fluorescently labeled (FL‐labeled) B‐Cur NPs or FL‐labeled S‐Cur NPs over time, while no obvious accumulation in tumors was discerned in the mouse injected with FL‐labeled curcumin (Figure 13d). Notably, the in vitro and in vivo biological assays demonstrated that S‐Cur NPs exhibited good biocompatibility and the highest anticancer activity (Figure 13e).

**Figure 14:**
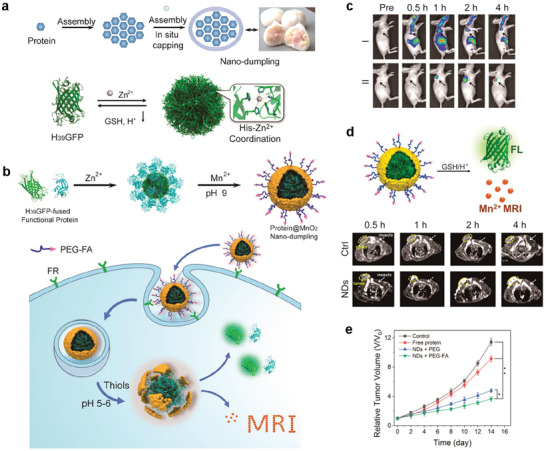
a) Protein preassembly strategy to design a dumpling‐like nanosystem for protein delivery and the scheme of the H_39_GFP and Zn^2+^‐induced self‐assembly of H_39_GFP into the nanocomplex. b) Schematic illustration of the efficient cancer cell targeting and FL/MRI bimodal visualized intracellular protein delivery by NDs. c) FL imaging of the free protein (I) and NDs (II). d) Scheme of the release of H_39_GFP and Mn^2+^ from NDs in the presence of GSH and low pH and the *T*_1_‐MR images for probing in vivo delivery of IRFP in the nude mice bearing a tumor (circled by yellow line) at different times after intravenous injection of protein‐free (Ctrl) and NDs. e) Tumor growth curves of the tumor xenografted mouse model after various treatments indicated. Reproduced with permission.^[^ ^76^ ^]^ Copyright 2020, American Chemical Society.

Zhu et al. prepared a protein@inorganic nanodumpling (ND) structure utilizing the Zn^2+^‐mediated assembly of protein with fused histidine‐rich assembling tags, followed by in situ biomineralization of MnO_2_, thus constructing an efficient protein delivery vehicle (NDs@PEG‐FA) to achieve ideal therapeutic efficacy (**Figure** **14**a,b).^[^ ^76^ ^]^ NDs@PEG‐FA possessed a high protein loading capacity (>63 wt%) and protein stability was significantly reinforced. Furthermore, biostimulation, including an intracellular high thiol content and acidic organelle environment, could induce protein release. Importantly, functional proteins were delivered to the tumor site in vivo, which was monitored through dual‐activatable FL/MR dual‐modal imaging (Figure 14c,d). Moreover, the ND system could be engineered to deliver therapeutic protein (i.e., RNase A) to the target tumor cells in a xenografted mouse model, effectively enhancing the tumor theranostic efficacy. Figure 14e confirmed that NDs@PEG‐FA had the highest antitumor potency. The results demonstrated that therapeutic proteins, such as natively histidine‐rich proteins and natively multicharged proteins, could be efficiently transported to the tumor sites by the protein‐preassembly‐dependent protein@inorganic nanosystem.

In a recent report, Shi et al. grafted the aggregation‐induced emission photosensitizer onto a phosphorothiolated DNAzyme backbone, which self‐assembled into nanoparticles with Zn^2+^ coordinated to the surface phosphorothioate group.^[^ ^77^ ^]^ When the obtained DNAzyme nanoparticles were located inside tumor cell lysosomes, ^1^O_2_ from photosensitizer could destroy the lysosome and promote the escape of Zn^2+^‐coordinated DNAzyme nanoparticles. As a result, the early growth response factor‐1 protein could be lowered by the hybrid DNAzyme nanoparticles, thus suppressing cancer cell growth and causing cancer cell apoptosis. Overall, considering the excellent biocompatibility of the Zn element in the human body and its importance in ensuring specific human functions, Zn^2+^ coordination can play a more compelling role in innovative modes to administer some anticancer drugs.

### 2.6 Ru^2+^‐Coordinated Self‐Assemblies

Today, Ru complexes, which possess several excellent properties, such as, low cytotoxicity, high activity in certain tumors, and impressive antimetastatic properties, are regarded as a new class of anticancer drugs. In the past two decades, some representative works focusing on supramolecular nanostructures based on Ru^2+^‐coordinated DNA have been reported, especially for cancer theranostic applications.^[^ ^79^, ^138^ ^]^ In particular, a growing number of metallacages and metalla‐rectangles have been constructed by Ru^2+^‐coordination‐driven self‐assemblies that show promising in vitro results as antitumor agents for human cancer cell lines.^[^ ^81^, ^139^ ^]^ For example, Adeyemo and coworkers prepared organometallic *η*6‐arene Ru^2+^ supramolecular architectures (MA_1_‐MA_4_) by the coordination‐driven self‐assembly of dinuclear Ru acceptors Ru_a_, Ru_b_, Ru_c_, and Ru_d_ separately with *N*,*N*,*N*′,*N*′‐tetra(pyridin‐4‐yl)‐[1,1’‐biphenyl]‐4.4’‐diamine in methanol.^[^ ^139f^ ^]^ The in vitro cytotoxicity of these four organometallic *η*6‐arene Ru^2+^ supramolecular architectures were studied, and the IC50 values indicated that the architectures were more cytotoxic than cisplatin against the tested cell lines. Additionally, Zhao et al. synthesized six tetranuclear rectangular metallacycles by the [2+2] coordination‐driven self‐assembly of imidazole‐based ditopic donor 1,4‐bis(imidazole‐1‐yl)benzene and 1,3‐bis(imidazol‐1‐yl)benzene with three analogous dinuclear Ru acceptors. Likewise, three hexanuclear trigonal prismatic metallacages were prepared via the [2+3] self‐assembly of tritopic donor 1,3,5‐tri(1*H*‐imidazol‐1‐yl) benzene with these Ru^2+^ acceptors. All the treated tumor cell lines (MCF‐7, HepG‐2, HCT‐116, HeLa, MDA‐MB‐231, and A549) were significantly inhibited by the prepared macrocycles and cages containing the 5,8‐dioxido‐1,4‐naphtoquinonato (donq) spacer, and decreased cytotoxicity was observed in HBE and THLE‐2 normal cells.^[^ ^81^ ^]^

Because there are more positive charges in multinuclear self‐assembled supermolecules, their solubilities are different from that of mononuclear complexes in lipids and water. Additionally, certain guest molecules can be accommodated in the internal cavity of the supermolecules acting as hosts; thus, cancer treatment drugs can be encapsulated to achieve controlled release in the cancer cell. For instance, utilizing the reactions of the dinuclear Ru^2+^ complex with the bidentate bridge ligand 4,4‐dipyridy and tridentate bridge ligand 2,4,6‐tris(pyridine‐4‐yl)‐1,3,5‐traizine, Yao et al. fabricated two kinds of Ru^2+^‐coordinated supramolecular complexes, hexa‐nuclear metallacage and tetranuclear metallacycle, via a self‐assembly supramolecular strategy under template‐free conditions. These two Ru‐based supramolecular complexes with a tunable host cavity can encapsulate one or two electron‐rich planar guests through *π*‐*π* stacking interactions.^[^ ^140^ ^]^

**Figure 15:**
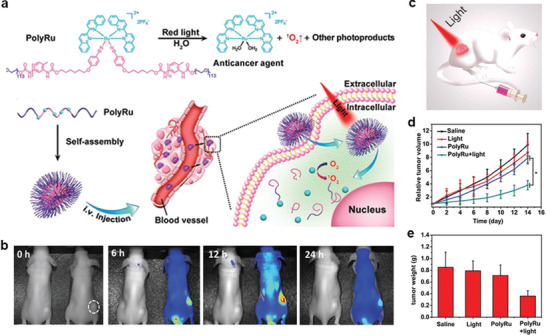
a) Structure of the amphiphilic polymetallodrug PolyRu. Red light induces the degradation of PolyRu to generate the anticancer complex [Ru(Biq)_2_(H_2_O)_2_](PF_6_)_2_ and ^1^O_2_ and schematic illustration of self‐assembly and phototherapy using PolyRu. b) In vivo fluorescence images of tumor‐bearing mice after intravenous injection of saline (left, control) and dye‐loaded PolyRu nanoparticles (right). c) Schematic illustration of anticancer phototherapy using PolyRu nanoparticles. Red light can activate the PolyRu nanoparticles accumulated at the tumor site. d) Relative tumor volume of tumor bearing mice during different treatments. e) Average weights of tumors at day 14 after treatment. Reproduced with permission.^[^ ^78^ ^]^ Copyright 2017, Wiley‐VCH.

The inability to accurately distinguish healthy cells from tumor cells is a major defect of anticancer drugs. Fortunately, photocaged Ru complexes are regarded as anticancer metallodrugs, which can enhance selectivity owing to their unique characteristics. For example, photocaged Ru complexes are usually nontoxic in tissues in the absence of laser irradiation, whereas they are toxic at the tumor sites upon laser irradiation. Moreover, photocaged Ru complexes can be activated by NIR light via a one‐photon process or a photon upconversion process, which subsequently generates singlet oxygen (^1^O_2_) for PDT and uncaged toxic Ru species or ligands from the complexes for photochemotherapy. Nevertheless, due to the small sizes, positive charges, poor hydrophilicity, and low biocompatibility, the in vivo applications of photocaged Ru complexes are still limited. To solve these issues, Sun et al. synthesized a novel Ru containing block copolymer (PolyRu) by a two‐step method; PolyRu self‐assembled into nanoparticles to act as a photoactivated polymetallodrug for combinatorial photochemotherapy and PDT (**Figure** **15**a).^[^ ^78^ ^]^ To validate the ability of PolyRu nanoparticles to accumulate in tumors, dye‐loaded PolyRu nanoparticles were intravenously injected into a HeLa tumor‐bearing mouse through the tail i.v. (Figure 15b). The maximum fluorescence intensity in the tumor occurred at 12 h of treatment. Furthermore, in vivo experiments (Figure 15c–e) in mice demonstrated that PolyRu efficiently accumulated at the tumor sites and inhibited the growth of tumors under red light irradiation.

**Figure 16:**
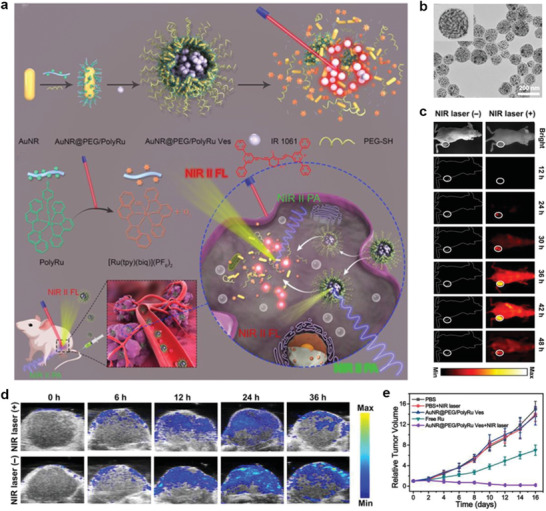
a) Schematic illustration of the preparation of NIR‐activatable AuNR@PEG/PolyRu vesicle. In vivo accumulation of the AuNR@PEG/PolyRu vesicle and disassociation of the nanoplatform after NIR irradiation, leading to sequential generation of ^1^O_2_ and release of chemotherapy drug Ru complex. b) TEM images of AuNR@PEG/PolyRu vesicle. c) In vivo NIR‐II FL images of the mice treated with AuNR@PEG/PolyRu vesicle and without or with laser irradiation. d) In vivo PA images of tumors in MCF‐7 tumor bearing mice treated with the AuNR@PEG/PolyRu vesicle at different post‐injection time points. e) Tumor growth curves of mice after intravenous injection of different formulations. Reproduced with permission.^[^ ^82^ ^]^ Copyright 2020, Ivyspring International Publisher.

The utilization of Ru‐coordinated complexes as photosensitizers for cancer theranostic has been studied by many other research teams. Ru complexes have drawn increasing attention as photosensitizers because their photochemical and biological properties can be adjusted with an appropriate choice of ligands for PDT.^[^ ^2a^ ^]^ For example, Song et al. prepared a dual NIR‐II PA and FL imaging vesicle nanoplatform by the self‐assembly of amphiphilic gold nanorod (AuNR) coated with a light‐responsive polyprodrug comprising a Ru complex (PolyRu) constructed via coordination bonds with the cyano groups of poly(6‐(4‐cyanophenoxy) hexyl methacrylate, a Ru complex [Ru(tpy)(biq)](PF_6_)_2_, PEG, and NIR‐II IR 1061 dye.^[^ ^82^ ^]^ The Ru complex acting as a photosensitizer and IR 1061 could be released from the AuNR vesicle upon sequential triggering under NIR light irradiation, resulting in a decrease in the NIR‐II PA signal and recovery of the NIR‐II FL signal. Furthermore, in addition to its chemotherapeutic capacity, the Ru complex generated ^1^O_2_ when exposed to NIR light irradiation (**Figure** **16**a).

The TEM images displayed in Figure 16b indicated that the AuNRs were closely attached to each other and formed a vesicular shell. An in vivo MFC‐7 tumor animal model was used to investigate the dual‐modal NIR‐II FL (Figure 16c) and PA (Figure 16d) imaging performance of the AuNR@PEG/PolyR vesicle. The results showed a gradual accumulation of the AuNR@PEG/PolyRu vesicle at the tumor site through the EPR effect, which could be slowly eliminated with time in the physiological environment. In addition, the AuNR@PEG/PolyRu vesicle was responsive to NIR light in vivo and the change in fluorescence signal after NIR laser irradiation could be used to monitor the release of the Ru complex and the disassembly process of the AuNR vesicle. In addition to the excellent in vivo responsive NIR‐II PA and FL imaging properties, AuNR@PEG/PolyRu combined with NIR laser irradiation also effectively inhibited tumor growth by synergistic chemotherapy and PDT (Figure 16e). The overall results indicated that the AuNR vesicle‐based system possessed not only precisely controlled drug release but also the intrinsic function to monitor active drug release and prodrug activation, thus providing a new concept for the development of cancer theranostic nanomedicines.

In addition to the works mentioned above, there are other research studies on Ru^2+^‐coordinated metallabowls,^[^ ^141^ ^]^ metallacycles,^[^ ^142^ ^]^ and metallogel.^[^ ^80^ ^]^ Overall, because of the intrinsic anticancer capability of Ru^2+^, Ru^2+^‐coordinated intelligent nanomedicine will attract more attention for the development of new cancer theranostic agents in the near future.

### 2.7 Rare‐Earth‐Coordinated Self‐Assemblies

Rare‐Earth (RE) elements possess different energy level configurations; thus, a variety of emission profiles can be achieved in RE‐based materials by choosing/combining suitable activators.^[^ ^17c^ ^]^ In recent decades, extensive efforts have been devoted to exploring nanoprobes with RE ions, including Nd^3+^, Yb^3+^, Pr^3+^ Sm^3+^, Dy^3+^, Ho^3+^, Er^3+^, and Tm^3+^, and using them for FL imaging. Specifically, the 15 lanthanide (Ln) elements belonging to the f‐block of the periodic table have the unique electronic configuration of [Xe] 4f*^n^*5d^1^6s^2^ (*n* = 0 (La) to 14 (Lu)). This unique electronic configuration, which combines the shielding of the 4f orbitals with the outer 5s^2^5p^6^ subshells, imparts Ln‐doped materials with unique magnetic and optical properties.^[^ ^143^ ^]^ With the exception of La^3+^, Gd^3+^, and Lu^3+^, the remaining Ln^3+^ ions possess sharp emission profiles in the NIR and/or VIS ranges, which are caused by intra‐configurational f‐f transitions. In addition, the forbidden nature of most of the f‐f transitions results in the long lifetimes of the Ln^3+^ ions (ns to µs for NIR and µs to ms for VIS emission). Importantly, the RE elements almost have no effects on the functions of proteins/enzymes in many physiological processes, including cell function promotion, immune system maintenance, and metabolism regulation.^[^ ^85^, ^144^ ^]^ Additionally, some lanthanides can inhibit the proliferation of cancer cells by facilitating the cellular uptake of certain drugs. Moreover, lanthanides can prevent the G0/G1 to S state transition, induce morphological changes, and trigger apoptosis through a receptor‐mediated extrinsic pathway.^[^ ^145^ ^]^

Some early reports focus on the construction of RE‐coordinated nanostructures with different compositions, morphologies, and applications.^[^ ^146^ ^]^ Subsequently, the development of nanomaterials for bioimaging^[^ ^85^, ^86^, ^90^, ^147^ ^]^ and cancer theranostics^[^ ^87^, ^144^, ^145^, ^148^ ^]^ attracted increasing research interest. Jin et al. reported an Nd integrated supramolecular polymeric nanoassembly to deliver siRNA and DOX for tumor therapy. In detail, polyethylenimine‐crosslinked‐g‐cyclodextrin (PC) was loaded with the adamantane modified DOX through the supramolecular assembly, thus constructing an interior DOX‐loaded PC (PCD). Subsequently, siRNA and PCD, driven by electrostatic interactions, self‐assembled into PCD/siRNA/Nd‐PC nanocomplexes.^[^ ^145^ ^]^

**Figure 17:**
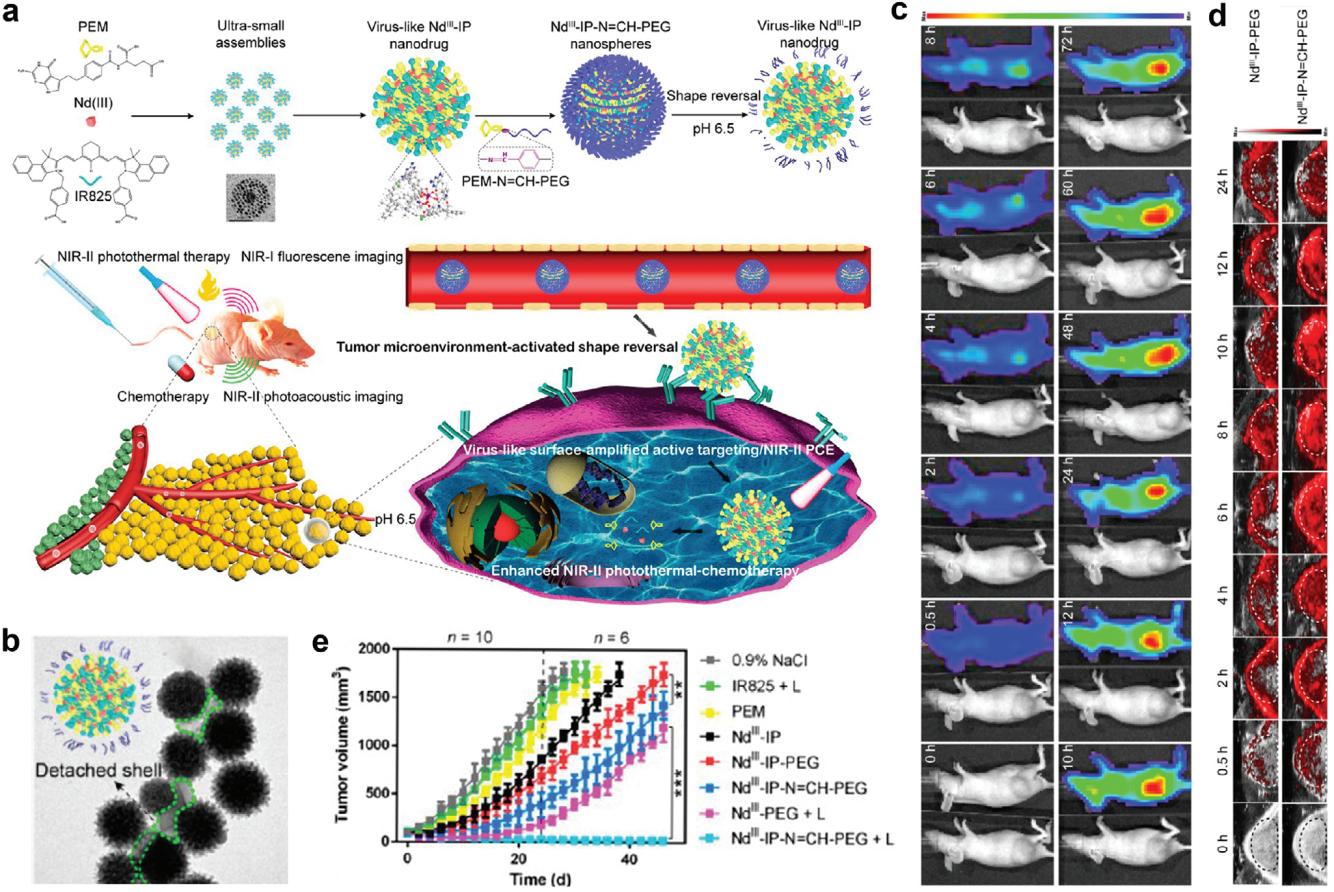
a) Illustration of the synthesis of Nd^3+^‐IP virus‐like nanodrug and tumor responsive shape‐reversal spherical‐shell camouflaged hierarchical Nd^3+^‐IP‐N = CH‐PEG and the tumor accumulation via reduced immune clearance and prolonged blood circulation. b) TEM image of Nd^3+^‐IP‐N = CH‐PEG at pH = 6.5. c) Long‐term time‐lapsed NIR‐I FL imaging of tumor‐bearing mice following injection of Nd^3+^‐IP‐N = CH‐PEG. d) NIR‐II PA imaging in tumors of tumor‐bearing mice following injection of Nd^3+^‐IP‐PEG and Nd^3+^‐IP‐N = CH‐PEG. e) Tumor volume change curves of tumor‐bearing nude mice. Reproduced with permission.^[^ ^83^ ^]^ Copyright 2019, American Chemical Society.

Li et al. synthesized an acidic TME‐responsive shape‐reversible metal‐organic virus‐inspired nanodrug containing a Nd^3+^ ion, NIR‐I‐emissive IR825, and a chemo‐drug (pemetrexed, PEM) via Nd^3+^‐coordination‐driven self‐assembly (**Figure** **17**a).^[^ ^83^ ^]^ A hierarchical nanoassembly was designed with a virus‐inspired core and camouflaged spherical shell to impart acidic TME‐responsiveness and shape‐reversibility, thus synergistically improving NIR‐II photothermal chemotherapy. Nd^3+^, as structural “transformers,” was introduced to adjust the structural geometry of IR825/PEM co‐assemblies, thus converting nanospheres to a virus‐like structure (Figure 17b). To obtain improved immune evading ability and prolonged circulation lifetime, the surface of a virus‐like structure was decorated with an acidic TME‐responsive PEG acting as a “shell,” thus ensuring ample accumulation in tumors. When the nanoassemblies were taken up into the tumors, the spherical shell fell off because of its responsiveness to the weakly acidic TME, thus realizing “sphere‐to‐virus” shape reversal; this property could improve the NIR‐II photothermal conversion efficiency, enhance cell adhesion, and activate tumor receptor‐mediated self‐targeting. In a single treatment cycle, the tumors were eliminated without regeneration through the TME specific enhanced NIR‐II photothermal chemotherapy guide by the FL/PA imaging capacity of the shell‐detached virus‐like nanodrug core (Figure 17c,d). As a result, when compared with the other groups, the mice treated with Nd^3+^‐IP‐N≡CH‐PEG and laser irradiation provided synergistic photothermal/chemotherapeutic effects to achieve the highest inhibition in tumor growth and greatly diminished immune clearance (Figure 17e).

**Figure 18:**
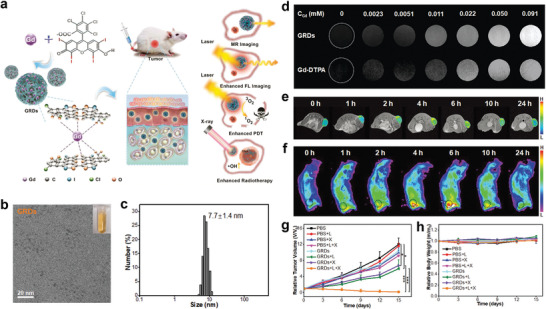
a) Schematic illustration for the preparation and in vivo FL‐/MR‐imaging‐guided PDT and radiotherapy of GRDs. b) TEM image and c) hydrodynamic diameter of GRDs. d) In vitro *T*_1_‐weighted MR images of GRDs and Gd‐DTPA. e) In vivo MR imaging of 4T1 tumors at different times post‐injection of GRDs intravenously (i.v.) and f) in vivo FL imaging of 4T1‐tumor‐bearing mice at different times post‐injection of GRDs i.v. g) Tumor growth curves of 4T1 tumors and h) relative change in mice body weights after various treatment. Reproduced with permission.^[^ ^89^ ^]^ Copyright 2020, Wiley‐VCH.

Sun et al. prepared Gd‐rose bengal (RB) coordination polymer nanodots (GRDs) utilizing the RB drug, a clinical photosensitizer, and Gd^3+^ by a solvothermal method (**Figure** **18**a).^[^ ^89^ ^]^ In a typical synthesis process, as the TEM test demonstrated, the GRDs were uniformly distributed and possessed an average size of 3.3 ± 0.8 nm (Figure 18b,c). Importantly, the FL intensity of the GRDs was 7.7‐fold that of RB, and the ^1^O_2_ generating capacity of the GRDs was 1.9‐fold that of RB. As a result, the GRDs were effectively used for MRI‐/enhanced‐FL imaging‐guided PDT and radiotherapy of cancers (Figure 18d–h). The GRDs+L+X treated groups, shown in Figure 18g, achieved the highest inhibition of tumor growth. In addition to this work, Zhao et al. developed a tumor‐targeted protein‐based probe for dual‐modal FL/MR imaging, which was synthesized by combining the target peptide (RGD) with an FL protein (RFP) and a small peptide (LBT) with a strong affinity for Gd^3+^ (RGD‐RFP‐LBT‐Gd).^[^ ^90^ ^]^

**Figure 19:**
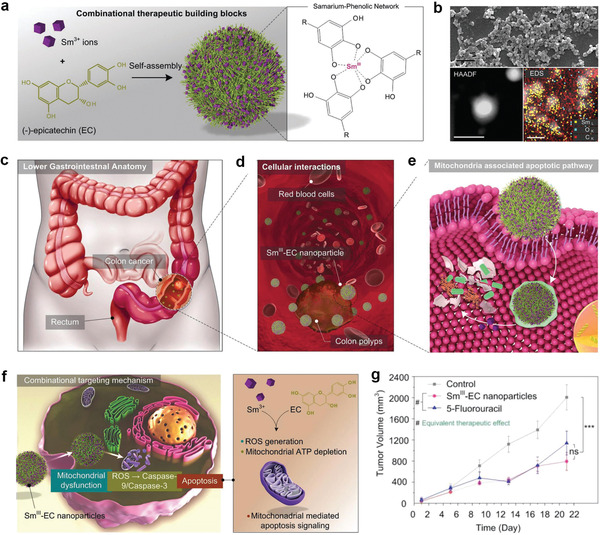
a) Self‐assembly of the Sm^3+^‐EC NPs from Sm^3+^ ions and phenolic EC molecules. b) SEM, EDS mapping, and HAADF images of Sm^3+^‐EC NPs. Scale bars are 500 nm in top panel of SEM image and 100 nm in EDS mapping and HAADF images. c) Overview of the lower gastrointestinal tract. d) Cellular interaction between colon polyps and Sm^3+^‐EC NPs. e) Intracellular delivery of functional Sm^3+^ ions and EC molecules through the endocytosis of Sm^3+^‐EC NPs. f) The possible mechanisms of Sm^3+^‐EC NPs‐induced toxicity on C26 cells. g) Tumor volume recorded at different time points. Reproduced with permission.^[^ ^84^ ^]^ Copyright 2019, Wiley‐VCH.

In 2009, Nishiyabu et al. developed nanoparticles with supramolecular networks self‐assembled in water from nucleotides with Ln^3+^ ions.^[^ ^147a^ ^]^ The prepared samples showed intrinsic functions, such as energy transfer from the nucleobase to Ln^3+^ ions and excellent performance as an MRI contrast agent. In another work, Li et al. developed an adenosine triphosphate depletion and ROS‐enhanced combined chemotherapy platform. Synergistic therapy was achieved via the mitochondrial dysfunction process depending on a metal‐phenolic network (MPN, Sm^3+^‐EC) constructed with the therapeutic Sm^3+^ ions and (‐)‐epicatechin (EC).^[^ ^84^ ^]^ The complex process between the Sm^3+^ ions and (‐)‐epicatechin (EC) is illustrated in **Figure** **19**a, and the images in Figure 19b depict the morphology and element composition of the formed Sm^3+^‐EC NPs. After the tumor cells take up Sm^3+^‐EC NPs through endocytosis, Sm^3+^ ions and EC, as therapeutic building blocks, can be released from the Sm^3+^‐EC NPs by degrading in response to the acidic TME (Figure 19c,d). As depicted in Figure 19e, Sm^3+^‐EC induces the apoptosis of colon cancer cells through the mitochondrial dysfunction process, leading to an excellent cancer treatment outcome. A possible mechanism of Sm^3+^‐EC nanoparticles‐induced toxicity on C26 cells is shown in Figure 19f. The apoptotic signal, such as an excessive ROS content, and the participation of caspase family proteins, are induced by the mitochondrial dysfunction process. PARP is activated by the activation of Caspase‐9, thus degrading Caspase‐3. Furthermore, the cleavage of caspase‐3 inactivates PARP, inducing programmed cell death. To assess in vivo antitumor activity, the authors made a comparison between the therapeutic efficacy of Sm^3+^‐EC NPs and that of the clinical anticancer drug 5‐fluorouracil. The tumor volume of Sm^3+^‐EC‐treated mice was nearly equal to that of the 5‐fluorouracil‐treated group and significantly smaller than that of the saline control group (Figure 19g). Importantly, Sm^3+^‐EC NPs did not influence the body weight of mice and normal organs, showing good biosafety.

**Figure 20:**
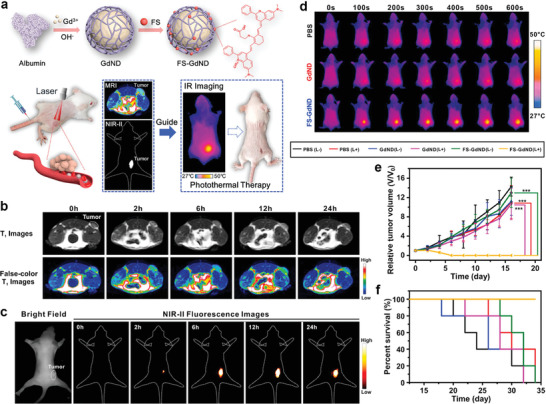
a) Schematic illustration of the synthesis of FS‐GdNDs and dual‐modal MR/NIR‐II imaging‐guided PTT mediated by FS‐GdNDs. b) *T*_1_‐weighted and false‐color mapped MR images of 4T1 tumor‐bearing mice preinjection and at predetermined time points post injection of FS‐GdNDs. c) Representative NIR‐II FL imaging of living mice at different time points after intravenous injection of FS‐GdNDs. d) Infrared thermography of 4T1 tumor‐bearing mice of the tumors after intravenous injection with PBS, GdNDs, and FS‐GdNDs under 808 nm laser irradiation. e) Time‐dependent tumor‐volume curves of mice in various groups. f) The survival curves of mice after different treatments. Reproduced with permission.^[^ ^149^ ^]^ Copyright 2020, Elsevier.

In a recent study, Li et al. utilized a small‐molecule NIR‐II fluorophore (FS) to decorate bovine serum albumin (BSA)‐mediated biomimetic mineralized gadolinium oxide nanodots (GdNDs), thus preparing ultrasmall versatile nanodots (FS‐GdNDs) for dual‐modal MR/NIR‐II imaging‐guided PTT (**Figure** **20**a).^[^ ^149^ ^]^ FS‐GdND was intravenously injected into inn4T1 tumor‐bearing mice, and the MRI signal of the tumor region at 2 h post injection was enhanced in comparison with the pre‐injection intensity (Figure 20b). Furthermore, to estimate the in vivo performance of NIR‐II FL, FS‐GdNDs was intravenously injected into nude mice bearing 4T1 tumors, which was imaged at different time points. Before injection, the background signal of VIS FL was hardly discernible, which indicated that the interference of autofluorescence was very weak. After injection, the tumor became bright gradually and the FL signal increased with time to peak at 12 h (Figure 20c). The in vivo infrared thermography capability of FS‐GdNDs was subsequently studied on 4T1 tumor‐bearing mice (Figure 20d). As shown, the tumor temperature in the FS‐GdNDs‐treated group increased continuously in the first 300 s and then plateaued at 50 °C, which indicated that a sufficiently enhanced temperature in the tumor region could kill tumor cells. However, the temperature of the tumor in the PBS and GdND‐treated groups increased negligibly. In vivo antitumor studies demonstrated that FS‐GdNDs successfully eliminated tumors and effectively enhanced the survival of mice during PTT (Figure 20e,f).

In a recent study, Liu et al. developed a self‐assembled metal‐phenolic nanocoating based on Gd^3+^ ions and catechin for the prevention of bacterial colonization.^[^ ^144a^ ^]^ By utilizing the long lifetime of Tb^3+^, an enzyme‐integrated coordination polymer composite was prepared and used for time‐resolved fluorescence detection of superoxide anions.^[^ ^144d^ ^]^ Recently, Zhou and coworkers developed a multi‐responsive Eu^3+^‐based hydrogel with shape‐memory, self‐healing, naked‐eye sensing, encryption, and antibacterial properties.^[^ ^144b^ ^]^ Through the coordination‐driven assembly of cypate and carboxyl ligand with Gd^3+^, Zhang et al. developed a novel NCP for multimodal imaging‐guided tumor‐targeting chemo‐PTT.^[^ ^88^ ^]^ Overall, due to the versatile physicochemical properties of the RE elements, RE‐coordinated supramolecular nanoconstructs are expected to find more practical applications in the future.

### 2.8 Pt(II)/(IV)‐Coordinated Self‐Assemblies

To date, a number of remarkable achievements have been reported for coordination‐driven self‐assembly, from accurate assemblies of various Pt metallacycles^[^ ^100^, ^102^, ^103^, ^104^, ^150^ ^]^ and metallacages^[^ ^104^, ^151^ ^]^ to multiple applications of these self‐assemblies, including cell imaging,^[^ ^152^ ^]^ optoelectronics, drug delivery,^[^ ^104c^ ^]^ and controlled release^[^ ^108^ ^]^ for cancer theranostic. Specifically, because of the different organic ligands and Pt‐based acceptors, the shape of the two‐dimensional Pt(II) metallacycles can be diverse, such as triangle,^[^ ^102^ ^]^ hexagon,^[^ ^153^ ^]^ rectangle,^[^ ^154^ ^]^ rhomboidal,^[^ ^104^, ^155^ ^]^ and other polygons.^[^ ^156^ ^]^ For example, Cook et al. reported the coordination‐driven self‐assembly of two highly emissive Pt(II) supramolecular triangle metallacycles containing a pyridyl‐functionalized BODIPY ligand.^[^ ^101^ ^]^ The Pt(II) ions served as the metal nodes of the triangles and provided the antitumor effect. The BODIPY cores within the triangles were delivered to the cancer cells, which could then be visualized using confocal laser scanning microscopy. Also, the BODIPY ligands acted as a photosensitizer for PDT. The integration of chemotherapy and PDT greatly improved anticancer efficacy through a synergistic therapeutic effect. Significantly, the ubiquitous issue of drug resistance was addressed. Yue et al. presented a nanodrug delivery platform constructed by self‐assembly based on metal coordination and covalent conjugation, which possessed a well‐defined size and geometry and high Pt(IV) loading efficiency.^[^ ^157^ ^]^ MTT and live/dead cell assays indicated that the supramolecular complexes exhibited superior therapeutic efficacy.

**Figure 21:**
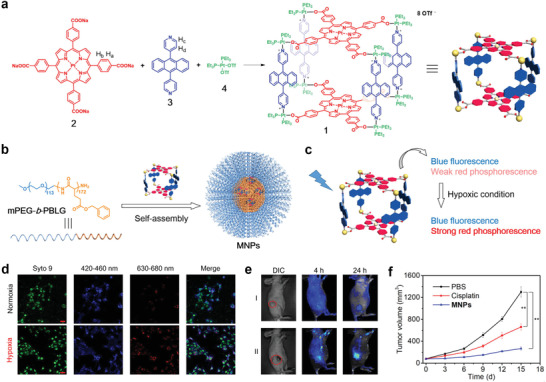
a) Chemical structures and cartoon representations of the preparation of 1, b) the formation of NPs, and c) the O_2_‐responsive emission. d) CLSM images of 4T1 cells stained by Syto 9 (green, staining nucleus) and MNPs (blue and red) under normoxic or hypoxic conditions. e) In vivo fluorescent images of different sized tumor‐bearing mice after the injection of MNPs. The signals were collected in the range of 600–800 nm with the excitation wavelength. f) Tumor volume changes of tumor‐bearing mice with different treatments during therapeutic period. Reproduced with permission.^[^ ^104d^ ^]^ Copyright 2020, Wiley‐VCH.

In a recent study, Zhu et al. first designed a Pt(II) metallacage (1) with dual‐emissive and phosphorescent properties^[^ ^104d^ ^]^ and then constructed metallacage‐loaded NPs (MNPs) by encapsulating (1) into the self‐assembled mPEG‐*b*‐PBLG through a co‐precipitation approach (**Figure** **21**a,b).^[^ ^104d^ ^]^ Based on the construction of metallacage, diverse ligands can be integrated through heteroligated coordinations, thus imparting the metallacage with dual‐emissive properties. In hypoxic conditions, the blue fluorescence of the metallacage was nearly unchanged, while the red phosphorescence was markedly enhanced (Figure 21c). After they were encapsulated by an amphiphilic polymer, the self‐assembled NPs were endowed with hypoxia imaging ability, which enhanced phosphorescence and lowered the intracellular O_2_ content (Figure 21d). Additionally, in vivo experiments revealed that the MNPs exhibited prolonged circulation life and high accumulation in tumors, which rendered them promising in clinical theranostic applications, including tumor hypoxia imaging and chemotherapy (Figure 21e). The tumor inhibition curves from antitumor assays, shown in Figure 21f, indicated that the inhibition capability of both PBS and cisplatin was lower than that of MNPs. These metallacages are highly sensitive, reliable as a hypoxia probe, and exhibit high chemotherapeutic efficacy and may therefore have wide cancer theranostic applications in the future.

Poulomi Sengupta et al. designed cholesterol‐succinic acid‐cisplatinum II‐based NPs from the cholesterol‐succinic acid‐platinum(II) molecule, phosphatidylcholine (PC), and 1,2‐distearoyl‐sn‐glycero‐3‐phosphoethanolamine‐*N*‐[amino(polyethylene glycol)‐2000] using a lipid‐film hydration self‐assembly method.^[^ ^95^ ^]^ The IC50 values of the prepared NPs were lower than those of carboplatin or cisplatin in vitro, and the NPs were still active under cisplatin‐resistant conditions. In addition, the NPs improved the antitumor effect in murine 4T1 breast cancer and K‐RasLSL/+/Ptenfl/fl ovarian cancer models with very low systemic and nephrotoxicity. Hou et al. utilized a bioinspired and biodegradable polymer mPEG‐b‐PpY to prepare two cis‐diamminedichloridoplatinum(II) (CDDP)‐loaded PpY/Pt and iPpY/Pt micelles, in which phosphato‐platinum bonds triggered supramolecular assembly, for systemic cisplatin delivery.^[^ ^96^ ^]^ Due to the phosphato‐platinum bonds, the micelles possessed relatively high stability in complicated physiological conditions, optimal sizes for nanodrugs, and high drug loading. Moreover, the micelles displayed faster drug release in response to pathological and intracellular signals, which could be utilized for on‐demand intracellular delivery. The experimental results indicated that both micelles exhibited prolonged circulation, improved accumulation in tumors, higher antitumor efficacy, and better tolerance than CDDP. For the 4T1 tumor model, iPpY/Pt was more effective than PpY/Pt in inhibiting tumor growth.

**Figure 22:**
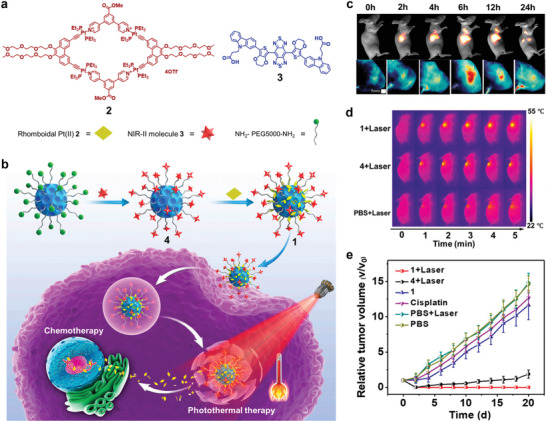
a) Structures of discrete Pt(II) metallacycle 2 and NIR‐II molecular dye 3. b) Schematic diagram of nanoagent 1 in chemophotothermal synergistic therapy. c) NIR‐II FL and PA images of U87MG tumor mice at different times after tail vein injection of 1. d) Infrared thermal images of mice treated with PBS, nanoagent 1, and 4 dots with laser irradiation. e) Relative tumor volumes for mice treated with different formulations. Reproduced with permission.^[^ ^104a^ ^]^ Copyright 2020, PNAS.

In 2019, Sun and coworkers developed melanin‐dot‐mediated Pt(II)‐coordinated supramolecular metallacycle complexes for chemo‐photothermal synergetic therapy under the guidance of PA and NIR‐II dual‐mode imaging.^[^ ^104a^ ^]^ The adopted dye (3) molecule with NIR‐II‐emissive property was synthesized from commercially available chemicals (**Figure** **22**a). Subsequently, the dye (3) molecule and a discrete Pt(II) metallacycle (2) were incorporated into the melanin dots to obtain a versatile theranostic nanoagent (1). In detail, the (4) dots were formed through the traditional coupling effect and then the Pt(II) metallacycle was loaded by *π*‐*π* binding. As a result, the theranostic probe (1) uniting the functions of imaging and therapy was successfully constructed (Figure 22b). The bioimaging property of the nanomedicine was verified, as shown in Figure 22c. As shown, the U87MG tumor‐bearing nude mice were i.v. injected with sample (1), and the PA and NIR‐II FL imaging signals of the live mice were recorded to study the in vivo dual‐modal imaging property. At time points 2, 4, 6, 12, and 24 h post‐injection, both the acquired PA and NIR‐II FL images could clearly distinguish the tumor from the surrounding background tissues. The chemo‐photothermal therapeutic efficacy of nanoagent (1) against U87MG tumors was investigated under laser irradiation at 808 nm. In Figure 22d, the temperature of tumor sites injected with (1) and (4) dots increased to ≈45.0 °C, whereas the mice treated with PBS and laser irradiation did not produce a discernible tumor temperature change. Interestingly, the tumor growths for the groups treated with cisplatin and 1 (single chemotherapy) were lower than that of the PBS and PBS‐plus‐laser treated groups (Figure 22e). Compared with cisplatin, the higher tumor inhibition capability of (1) was attributed to the nanoparticle‐mediated EPR effect. For the mice treated with (1) plus laser (chemo‐PTT) and 4 dots plus laser (single PTT), the corresponding tumor growths were markedly inhibited. However, incomplete ablation of the tumorous cells treated by single PTT led to tumor recurrence after 12 days.

**Figure 23:**
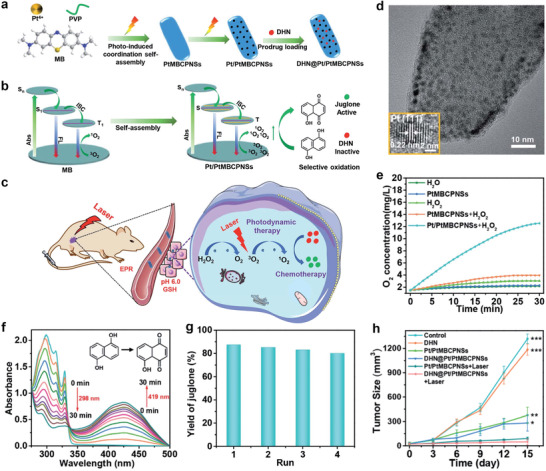
a) Schematic illustration for the preparation of the Pt/PtMBCPNSs and loading of prodrugs. b) Enhanced ^1^O_2_ production and selective oxidation of DHN. c) The application of Pt/PtMBCPNSs in PDT and chemotherapy. d) TEM images of Pt/PtMBCPNSs. e) The amounts of O_2_ produced using various systems (H_2_O, Pt/PtMBCPNSs, H_2_O_2_, PtMBCPNSs + H_2_O_2_, and Pt/PtMBCPNSs + H_2_O_2_) over time. f) UV–vis absorption spectra for DHN in PBS solution with Pt/PtMBCPNSs and subjected to photooxidation for various durations. g) Yields of juglone for various numbers of cycles. h) Tumor sizes of tumor‐bearing mice after various treatments. Reproduced with permission.^[^ ^105^ ^]^ Copyright 2020, the Royal Society of Chemistry.

In a recent work, He et al. prepared a Pt(IV) methylene blue coordinated polymer (PtMBCP), and the size‐tunable and porous Pt NP/PtMBCP nanoshuttles (Pt/PtMBCPNSs) were fabricated via sequential topotactic conversions to achieve in vivo drug synthesis and cancer treatment (**Figure** **23**a–c).^[^ ^105^ ^]^ As shown in Figure 23d, the TEM image of Pt/PtMBCPNSs indicated that the Pt NPs were uniformly distributed on the obtained nanoshuttles. The lattice spacing of the nanocrystal was determined to be 0.22 nm, which matched the spacing of the Pt (111) crystal lattice. The O_2_ generating ability of Pt/PtMBCPNSs was also investigated. As shown in Figure 23e, rapid O_2_ generation was observed when the Pt/PtMBCPNSs were dispersed in a solution of H_2_O_2_. However, O_2_ was hardly generated in the presence of PtMBCPNSs alone. Therefore, Pt/PtMBCPNSs effectively mitigate hypoxia in the TME and therefore enhance the PDT efficacy because they catalyze the decomposition of H_2_O_2_ into O_2_.

Notably, ^1^O_2_ plays a key role in the highly selective photooxidation of 1,5‐dihydroxynaphthalene (DHN), whose reaction product, juglone, widely serves as an antitumor drug. Therefore, Pt/PtMBCPNSs were used to catalyze the oxidation of DHN to generate juglone. As shown in Figure 23f, under laser irradiation at 660 nm, the absorption intensity of juglone at the wavelength of 419 nm increased, while that of DHN at 298 nm decreased. The yield of juglone was calculated to be 87.23% and the catalyst showed good reusability (Figure 23g). The in vivo antitumor results are shown in Figure 23h in which the results of six groups are compared. Significant tumor inhibition was observed at the tumor site of the mice injected with DHN@Pt/PtMBCPNSs and exposed to laser irradiation, and after 15 days of treatment, the growth of 95.5% of these tumors was suppressed. During the entire therapy course, the main organs of the mice were undamaged, which demonstrated the excellent biosafety of DHN@Pt/PtMBCPNSs.

Because of the unique in situ nontoxic to toxic antitumor mechanism and the good coordination of the Pt ion, many other nanostructures have been constructed and studied for cancer theranostic applications.^[^ ^97^, ^98^, ^99^, ^106^, ^107^, ^158^ ^]^ For future anticancer research work, a possible avenue to enhance cancer theranostic efficacy might entail tumor‐specific Pt release and induced formation of self‐assembled nanostructures with damage capability to cell DNA.

### 2.9 Other Metal‐Coordinated Self‐Assemblies

In addition to the abovementioned metal ions, other metal ions have also been studied for the construction of coordinated nanoassemblies for cancer theranostic applications. For example, palladium (II) complexes are possible analogs of anticancer platinum complexes because of their similar tetradentate square‐planar structure and d^8^ coordination sphere. Thus, various palladium metal supramolecules with barrel,^[^ ^159^ ^]^ rod,^[^ ^160^ ^]^ polyhedron,^[^ ^161^ ^]^ cage,^[^ ^162^ ^]^ and other architectures have been constructed by coordination‐driven self‐assembly for cancer theranostic applications. Furthermore, optimization/fine‐tuning of their properties is achieved by utilizing various functional ligands, such as BODIPYs, a family of fluorescent dyes. For example, Gupta et al. synthesized four palladium‐coordinated supramolecules with triangular/square architectures from boron dipyrromethane by coordination‐driven self‐assembly.^[^ ^163^ ^]^ These obtained supramolecules exhibited higher toxicity to glioblastoma cancer cells than cisplatin and negligible toxicity to normal lung fibroblasts. The green emission of the BODIPY ligands in these supramolecules observed with a confocal microscope indicated that the compounds were localized in the cytoplasm and on the plasma membrane.

In recent years, gold (Au)‐based NPs have attracted increasing interest in targeted delivery, bioimaging, theranostic, and biosensing fields. For instance, Yan et al. synthesized an injectable and self‐healing collagen‐protein‐based hydrogel by mixing an acidic collagen aqueous solution with HAuCl_4_ solution under ambient conditions via Au‐biomineralization‐triggered self‐assembly, mainly due to the electrostatic interaction between [AuCl_4_]^−^ ions and positively charged collagen chains.^[^ ^164^ ^]^ The prepared Au NPs were demonstrated to tune the mechanical nature of collagen‐based hydrogels, in which the reversible electrostatic and/or coordination interaction between the Au NPs and collagen chains imparted the hydrogels with self‐healing and shear‐thinning capabilities. As a result, the antitumor effect of the developed hydrogel was markedly enhanced through combined PTT and PDT. Other works have been reported on the use of the coordination effect between Au and organic ligands to prepare functional nanohybrids.^[^ ^59^, ^165^ ^]^

Additionally, some studies focus on exploiting the advantages of metal‐coordinated self‐assembling strategies to improve cancer therapy efficacy. For example, a metallopolymer micelle with NIR‐controlled HER‐2‐targeting capability was invented for integrated PDT and chemotherapy on HER‐2‐overexpressed cancer.^[^ ^166^ ^]^ The multifunctional micelle was formed by polymerizing cyclometallated Ir^3+^ complex possessing non‐emissive ^3^IL state with lapatinib‐conjugated PEG monoether and then co‐assembling with upconversion NPs. Zhao et al. fabricated a supramolecular porphyrin nanotube to promote the production of ^1^O_2_ for PDT by using out‐of‐plane coordinated Bi^3+^‐porphyrin, which displayed J‐aggregation.^[^ ^167^ ^]^ Moreover, utilizing the interfacial coordination interactions between the Ag^+^ ion at the surface of Ag NPs and O/N atom of a fluorescent cyano‐carboxylic derivative (noted as CECZA), a two‐photon PTT agent was fabricated through self‐assembly of Ag NPs and CECZA, and the assemblies demonstrated good PTT effect on HeLa cells.^[^ ^168^ ^]^ Apart from the works mentioned above, there are others studies on the use of the coordination effect derived from V^3+/4+^,^[^ ^24^, ^169^ ^]^ Zr^4+^,^[^ ^170^ ^]^ Ag^+^,^[^ ^171^ ^]^ Hf^4+^,^[^ ^91^, ^92^, ^93^, ^94^ ^]^ Sn^4+^,^[^ ^172^ ^]^ Re^+^,^[^ ^173^ ^]^ Ir^3+^,^[^ ^174^ ^]^ and Bi^3+[^ ^175^ ^]^ to fabricate various nanoplatforms for cancer theranostic investigation.

### 2.10 Multiple‐Coordinated Self‐Assemblies

Through good design, multimetal‐coordinated supramolecular nanosystems can achieve ideal theranostic outcomes due to the synergistic effect of multimetal ions and multi‐coordinated components;^[^ ^176^ ^]^ thus, the advantages of various metal ions and ligands are fully exploited. During the past two decades, there are some studies have focused on exploring multimetal‐coordinated nanostructures.^[^ ^15^, ^16^, ^17^, ^177^ ^]^ Su et al. have devoted a considerable amount of effort to the study of multimetal coordination supramolecular self‐assemblies. In the early stage, stereochemically stable D3‐symmetry [Ru(phen)_3_]^2+^ type metal‐organic ligands were used to realize the self‐assembly of monochiral coordination molecular cages through chiral recognition and transfer, and a pair of metal‐enantiomers of metal‐organic cages was obtained.^[^ ^178^ ^]^ Furthermore, by exploiting the high thermodynamic stability of the octahedral chiral three‐dimensional metal center and by using the method of pre‐splitting the secondary structural element precursor and step‐by‐step assembly, a pure chiral metal‐organic molecular cage was successfully obtained, and the C2‐chiral separation of enantiomers of symmetric organic small molecules was achieved.^[^ ^179^ ^]^ Moreover, by using the chiral coordination space of the molecular cage, the regio and stereoselective photocatalytic coupling reaction was successfully realized.^[^ ^180^ ^]^ Based on the above results, they recently reported the coordination‐driven self‐assembly of chiral molecular cages to Fe^2+^ and successfully obtained high‐purity monochiral Fe‐Pd bimetallic coordination molecular cages using a stepwise self‐assembly method.^[^ ^176d^ ^]^ Along with the development of the multimetal coordination technique, other studies on the use of multimetal‐coordinated nanoconstructs for bioimaging,^[^ ^181^ ^]^ cancer diagnosis and/or therapy,^[^ ^109^, ^182^ ^]^ and other biomedical applications^[^ ^176^, ^183^ ^]^ have been reported.

**Figure 24:**
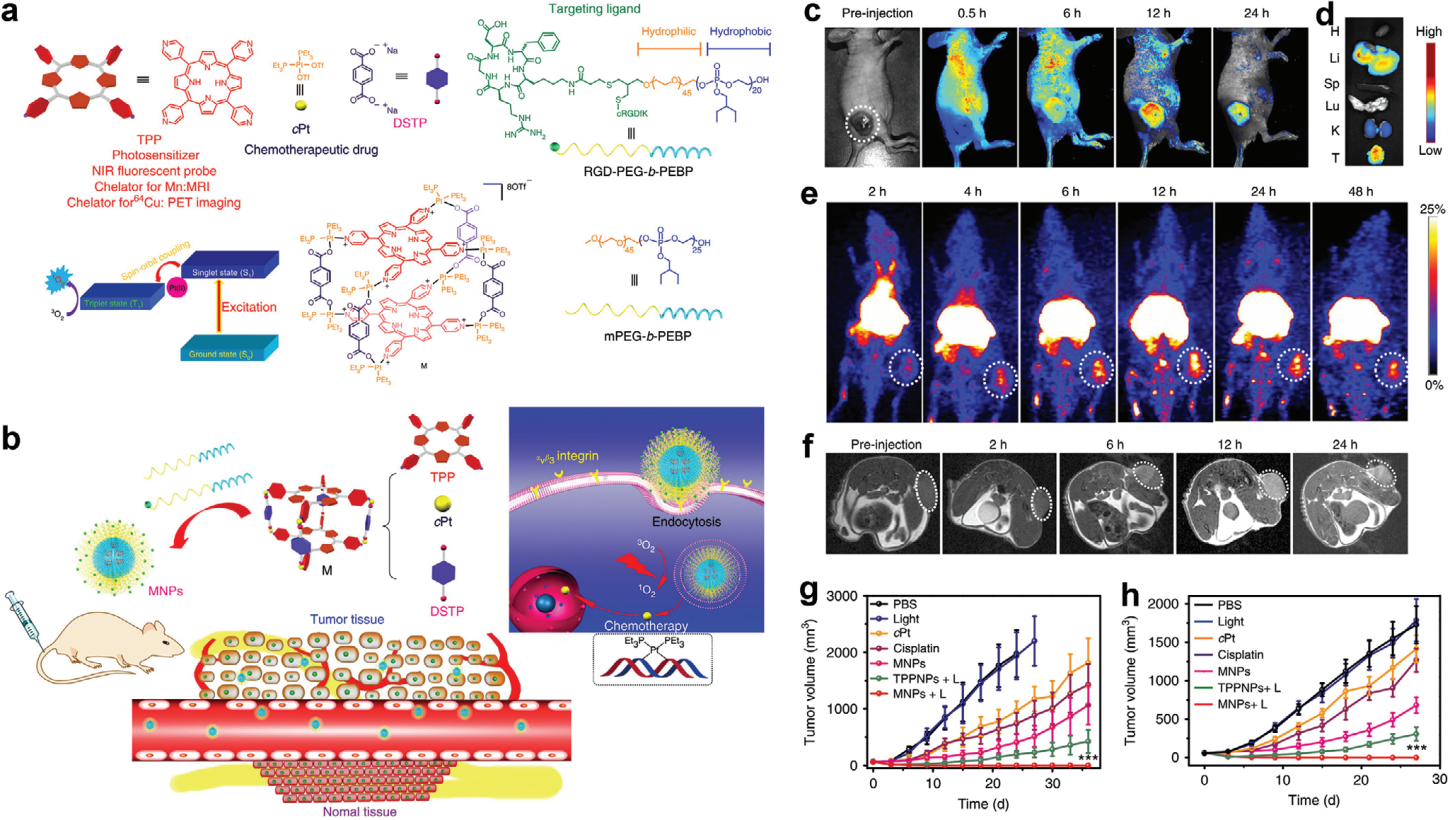
a) Structures of TPP, cPt, DSTP, M, mPEG‐b‐PEBP, and RGD‐PEGb‐PEBP. b) Schematic illustration of MNPs accumulation in tumor tissue followed by EPR effect and receptor‐mediated endocytosis, and their applications in subcutaneous (U87MG), drug‐resistant (A2780CIS), orthotopic (4T1 and LM3) tumor treatments, and lung anti‐metastasis. c) NIRFL imaging of U87MG tumor‐bearing mice following i.v. injection of MNPs. d) Ex vivo image of the main organs separated from tumor‐bearing mice at 24 h post injection of MNPs. e) PET image of tumor‐bearing mice at different time points post injection of ^64^Cu@MNPs. f) In vivo *T*_1_‐weighted axial MR images of the mice preinjection and after injection of Mn@MNPs. g) Tumor growth curves for the mice after different formulations for U87MG. h) In vivo tumor growth inhibition curves for A2780CIS. Reproduced with permission.^[^ ^110^ ^]^ Copyright 2018, Springer Nature.

As a typical example, Yu et al. developed an organoplatinum (II) metallacage (M) via multiple coordination‐driven self‐assembly utilizing 5, 10, 15, 20 tetra(4‐pyridyl)porphyrin (TPP), disodium terephthalate, and cis‐(PEt_3_)_2_Pt(OTf)_2_(cPt) as the building blocks to achieve active targeting ability and improved EPR effect (**Figure** **24**a).^[^ ^110^ ^]^ The EPR effect‐mediated accumulation of MNPs in tumors, receptor‐mediated endocytosis, and therapy application are illustrated in Figure 24b. The NIR optical images, displayed in Figure 24c, indicated significant accumulation of the MNPs in the tumors of mice 6 h post injection, and a stronger fluorescence signal was detected in the tumor site 24 h post injection compared with other tissues. E*x vivo* NIRFL imaging of isolated organs was tested 24 h post injection, and the corresponding optical images are displayed in Figure 24d. As shown, the heart, spleen, and lung exhibited very low fluorescence, while the excised tumor tissue showed notable fluorescence. Furthermore, in vivo PET and MR imaging were successfully conducted to track the delivery and biodistribution of ^64^Cu@MNPs in a tumor‐bearing mouse (Figure 24e,f). Overall, the trimodal imaging function of the MNPs allowed efficient diagnosis of the tumor and real‐time monitoring of the delivery, biodistribution, and excretion of the MNPs. Significantly, the MNPs exerted antimetastatic effect and superior antitumor effect against all kinds of drug‐resistant A2780CIS and orthotopic and U87MG tumor models, and the tumors were ablated without recurrence after a single therapy (Figure 24g,h).

**Figure 25:**
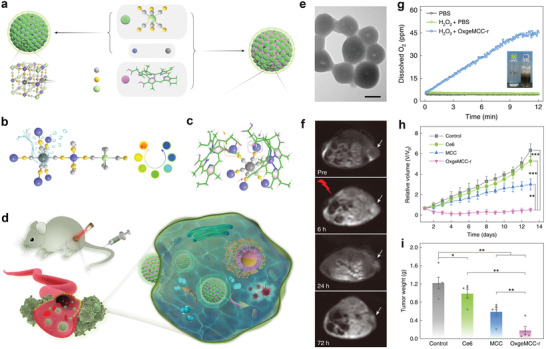
Schematic illustration of OxgeMCC‐r. a) OxgeMCC‐r consists of catalytically active single‐atom Ru site anchored in MCC with outer PVP protection layer. b) Partial molecular structure of OxgeMCC‐r with active singleatom Ru site serving as catalase‐like nanozyme for O_2_ generation. c) Multicomponent coordination interactions within the OxgeMCC‐r SAE. d) Scheme of continuously catalytic O_2_ generation and ROS production for enhanced PDT by OxgeMCC‐r SAE. e) TEM images of OxgeMCC‐r. f) MR imaging of tumor‐bearing mouse at different treatment points. g) O_2_ generation after treating OxgeMCC‐r SAE with H_2_O_2_ in PBS. Inset is a photograph of H_2_O_2_ solutions in the presence or absence of OxgeMCC‐r SAE. h) Relative tumor volumes of mice after various treatments. i) Average weights of tumors from different groups of mice after various treatments. Reproduced with permission.^[^ ^111^ ^]^ Copyright 2020, Springer Nature.

In 2018, Wang et al. reported the development of a PDT nanoagent named OxgeMCC‐r single‐atom enzyme (SAE), which consisted of single‐atom Ru anchored in a metal‐organic framework Mn_3_[Co(CN)_6_]_2_ with Ce6 encapsulation (**Figure** **25**a–d).^[^ ^111^ ^]^ Utilizing Mn_3_[Co(CN)_6_]_2_ as the support material, single‐atom Ru was incorporated into the framework to partially substitute Co and to act as a catalytic site for endogenous O_2_ generation. Facilitated by the coordination effect and other supramolecular interactions, metal ions, Ce6, and organic ligand encapsulated by PVP polymer could self‐assemble to form the uniform and well‐defined OxgeMCC‐r SAE (Figure 25e). The images displayed in Figure 25f indicated that OxgeMCC‐r SAE can be used as an MRI agent. OxgeMCC‐r SAEs enhanced the sensitivity of *T*_1_‐weighted MRI owing to the existence of high‐spin Mn‐N_6_ (S = 5/2) species, which permitted in vivo tracking of the therapeutic nanosystem. The loading of Ce6 in OxgenMCC‐r SAE was increased because of the intrinsic porous property of the metal‐organic framework. High catalytic durability and ability were ensured by the rapid generation of O_2_ from endogenous H_2_O_2_ without external activation or self‐consumption (Figure 25g). The high catalytic activity of this nanozyme was attributed to the six unsaturated Ru‐C6 coordination sites, which resulted in the rapid decomposition of H_2_O_2_ to alleviate tumor hypoxia. Significantly, the mice injected with OxgeMCC‐r SAEs showed remarkable tumor suppression upon laser irradiation (Figure 25h,i).

## 3 Conclusion and Outlook

In this review, recent advancements on metal‐coordinated supramolecular nanoassemblies based on organic ligands for cancer theranostic applications were summarized. The formation mechanisms of these nanoassemblies relied on the coordination effect between the functional groups of organic molecules and the metal ions in different conditions. Because of the numerous choices in organic ligands and metal ions, a number of coordinated supramolecular nanoassemblies have been investigated as nanomedicines for cancer theranostic applications.^[^ ^6^, ^74^, ^184^ ^]^ The metal‐organic coordination endowed the nanomedicines with several advantageous properties for cancer theranostics, including multiple functions, prolonged blood circulation, tailored drug release, accurate bioimaging, and superior anticancer performance. Specifically, TME‐responsive nanocarriers which permitted the targeted release of metal ions and high loading of functional ligands enabled the development of intratumoral metal‐coordinated self‐assembling supramolecular nanomedicines, which are highly promising in solving the intractable problem of low accumulation of anticancer components in tumors.

In addition to the adjustable structures and physicochemical properties, good biocompatibility of the coordinated supramolecular nanomedicines can be also enabled by utilizing alternative raw materials and components with hypotonicity or nontoxicity. Further functionalization of the components renders the obtained nanomedicines biodegradable and clearable, which can greatly improve their biosafety. The facile surface modification of coordinated nanomedicines by using biomolecules that target molecules or proteins ensures good biosecurity and relatively high accumulation in tumors, thereby realizing improved theranostic outcomes.^[^ ^185^ ^]^ Additionally, the high loading capacity of metal‐coordinated supramolecular nanomedicines can be beneficial for delivering all kinds of molecules, including imaging agents, detection probes, and therapeutic drugs. Overall, metal‐coordinated supramolecular nanomedicines are exceptionally promising in bridging the obvious boundary between inorganic and organic anticancer drugs; thus, they can integrate multimodal diagnosis and therapy and ultimately improve the efficacy of cancer theranostic strategies.

Although the prospects are very promising for cancer theranostic strategies, some crucial technology concerns and challenges persist, especially in the development of metal‐coordinated supramolecular nanomedicines. First, from the metal ion perspective, more attention should be paid to the mechanism of metal‐coordinated supramolecular nanotheranostics; thus, choosing bioactive and reliable metal ions is crucial for in vivo applications. Second, the challenges of cancer therapeutic systems from the complexity and heterogeneity of the tumor entity and TME include incomplete prediction of the interactions (fluid pressure, trafficking, penetration, internalization, distribution) between the nanotherapeutic and the tumor cell. A profound understanding of tumor biology, noninvasive bioimaging techniques with ultrahigh resolution, and rapid response at the single‐cell level should clarify the complicated interactions between the theranostic platform and the tumor. Third, the stability of metal‐coordinated nanomedicines is another major concern. Metal‐coordinated supramolecular self‐assembly occurs through noncovalent interactions; thus, the nanomedicines may decompose in complicated physiological microenvironments, which can lead to early drug release and side effects to normal tissues. Particularly, when exploiting intratumoral coordinated self‐assembling nanoformulations, nano reagents with high stability in blood circulation and instability in tumor sites can be considered ideal candidates. Considering the possible interference of the physicochemical characteristics of the TME on the intratumoral metal‐coordinated self‐assembly processes, TME modulation caused by the fabricated nanoconstruct should be well‐controlled. Overall, through optimization of structures and properties, metal‐coordinated supramolecular nanotheranostic strategies are very effective and indispensable in the development of next‐generation cancer theranostic nanomedicines.

## Conflict of Interest

The authors declare no conflict of interest.
